# COVID-19 associated thyroid dysfunction and other comorbidities and its management using phytochemical-based therapeutics: a natural way

**DOI:** 10.1042/BSR20230293

**Published:** 2023-07-26

**Authors:** Arpana Parihar, Shivani Malviya, Raju Khan, Ajeet Kaushik, Ebrahim Mostafavi

**Affiliations:** 1 Industrial Waste Utilization, Nano and Biomaterials, CSIR-Advanced Materials and Processes Research Institute (AMPRI), Hoshangabad Road, Bhopal 462026, MP, India; 2 Department of Biochemistry and Genetics, Barkatullah University, Habib Ganj, Bhopal, Madhya Pradesh 462026, India; 3 Academy of Scientific and Innovative Research (AcSIR), Ghaziabad 201002, India; 4 NanoBioTech Laboratory, Department of Environmental Engineering, Florida Polytechnic University, Lakeland, FL 33805, U.S.A.; 5 School of Engineering, University of Petroleum and Energy Studies, Dehradun 248007, India; 6 Stanford Cardiovascular Institute, Stanford University School of Medicine, Stanford, CA 94305, U.S.A.; 7 Department of Medicine, Stanford University School of Medicine, Stanford, CA 94305, U.S.A.

**Keywords:** COVID-19, Phytochemicals, Post-COVID-19 complications, SARS-CoV-2, Thyroid Function

## Abstract

The present severe acute respiratory syndrome-2 (SARS-CoV-2) mediated Coronavirus pandemic (COVID-19) and post-COVID-19 complications affect human life drastically. Patients who have been cured of COVID-19 infection are now experiencing post-COVID-19 associated comorbidities, which have increased mortality rates. The SARS-CoV-2 infection distresses the lungs, kidneys, gastrointestinal tract, and various endocrine glands, including the thyroid. The emergence of variants which includes Omicron (B.1.1.529) and its lineages threaten the world severely. Among different therapeutic approaches, phytochemical-based therapeutics are not only cost-effective but also have lesser side effects. Recently a plethora of studies have shown the therapeutic efficacy of various phytochemicals for the treatment of COVID-19. Besides this, various phytochemicals have been found efficacious in treating several inflammatory diseases, including thyroid-related anomalies. The method of the phytochemical formulation is quick and facile and the raw materials for such herbal preparations are approved worldwide for human use against certain disease conditions. Owing to the advantages of phytochemicals, this review primarily discusses the COVID-19-related thyroid dysfunction and the role of key phytochemicals to deal with thyroid anomaly and post-COVID-19 complications. Further, this review shed light on the mechanism via which COVID-19 and its related complication affect organ function of the body, along with the mechanistic insight into the way by which phytochemicals could help to cure post-COVID-19 complications in thyroid patients. Considering the advantages offered by phytochemicals as a safer and cost-effective medication they can be potentially used to combat COVID-19-associated comorbidities.

## Introduction

Severe acute respiratory syndrome-2 mediated Coronavirus illness (COVID-19) has triggered a global catastrophe that has impacted several aspects of human life. SARS-CoV-2 had evolved over the past 2 years and a variety of mutant variants have emerged [1,2,279]. These variants have been classified into three categories: variants of interest (VOIs), variants of concern (VOCs), and variants under monitoring (VUMs). Further, the VOCs are categorized based on mutations as Alpha (B.1.1.7), Beta (B.1.351), Gamma (P.1), and Delta (B.1.617.2). Another variant named Omicron (B.1.1.529) identified on November 26, 2021, was nominated as the fifth VOC by WHO, and gained worldwide attention due to its high transmissibility rate and varied symptoms among the population [3–6]. The world is under constant threat due to the appearance of variants of SARS-CoV-2 and COVID-19-associated complications. Hence, the role of diagnostics [2,7–18] and therapeutic [13,19–21,278] modalities in this regard are crucial in the management of viral infections. The virus mainly targets the lungs but along with it, other organs such as the kidneys, gut, and various endocrine glands such as the thyroid gland also get affected due to COVID-19-related post-complications [1]. Multiple organ failure and a variety of clinical symptoms result from the virus's ability to spread throughout the body. This feature of SARS-CoV-2 has also been seen in other viruses too such as in the case of the hepatitis C virus [22].

Virus infections are key environmental variables in the pathogenesis of certain forms of thyroid abnormalities, such as autoimmune thyroid illnesses and subacute thyroiditis. There is evidence that thyroiditis can occur in response to viral infection. Several studies, such as the presence of virus particles in patients' thyroid glands and an increase in anti-virus antibodies in people with thyroid problems, corroborate this hypothesis. Even though SARS-CoV-2 has distinct traits that set it apart from other viruses, research suggests that divergent viruses use similar methods to infect the host [23]. Viruses are hypothesized to play a crucial role in the onset and progression of diseases in a variety of ways. Some viruses cause host cell death and release cellular antigenic components which further led to the inflammatory response. Another mechanism by which the virus elicits an antiviral response is that it targets autoantigens as molecular mimicry. It has also been discovered that abnormal cytokine and chemokine production and release could lead to abnormal expression of MHC class II molecules and activation of Toll-like receptors that play a foremost role in the pathogenesis of human viral infections [8,9]. As of August 26, 2022, approximately 596,873,121 confirmed cases of COVID-19 have been reported including 6,459,684 deaths, globally [24]. COVID-19 is the next millennium's pandemic, posing significant global health challenges [25]. SARS-CoV-2 is a novel strain having enveloped RNA β- coronavirus [26]. SARS-CoV-2 is phylogenetically related to SARS-CoV-1, the causative agent of SARS [12–14] SARS-CoV-2, like SARS-CoV-1, infects humans via angiotensin-converting enzyme 2 (ACE2) receptor [27,28].

Coronaviruses consist of a protease enzyme which is essential for the generation of viral proteins as well as the regulation of the activity of enzymes involved in viral replication. Coronavirus infection causes various manifestations in human body, ranging from asymptomatic infections to the common cold to more serious and even fatal damage to respiratory organs which could include acute respiratory distress syndrome [29]. The enhanced inflammatory response resulted in a cytokine storm due to SARS-CoV-2 infection affecting various organs including endocrine glands such as the thyroid. In this context, the thyroid hormones and signaling molecules which act as immunomodulators are involved in a complicated interplay that aggravates virus infection. The scientific evidence has demonstrated this fact in both pathological and physiological contexts [30,31]. Viral infection followed by inflammatory-immune reactions was found to be a primary determinant that affects lifelong thyroid function, evaluation of this could help to determine the individual’s ‘thyroid biography’ [32]. The inadequacy of targeted medicines and vaccines, together with the appearance of mutant variants of SARS-CoV-2 which causes enhanced COVID-19-related complications forced scientists to look for novel antiviral formulations which can not only target the viral infection but also take care of post-COVID-19 complications [33]. In the past, epidemics of MERS-CoV and SARS-CoV have instilled us information on the antiviral efficacy of several phytochemicals with aided health advantages. The phytochemical-based therapeutics are not only cost-effective but also have lesser side effects.

Recently several studies have shown the therapeutic efficacy of various phytochemicals-based formulations for the treatment of COVID-19 [34–37] which we will discuss in a later section. Polyphenols originating from plants have been employed as pharmaceutical formulations and can be taken as functional foods. The way of executing such formulations is quick due to the ease of availability of raw materials for such preparations. These herbs and consumable plants are not only easily available but also permitted for human use worldwide. Besides, various phytochemicals have been found efficacious in treating various inflammatory diseases including thyroid-related anomalies [38–40]. Owing to the advantages of phytochemicals, this review primarily discussed COVID-19-related thyroid dysfunction and the role of key phytochemicals to deal with thyroid anomalies and post-COVID-19 complications. There are several other reviews that either deal with the importance of phytochemicals or discuss post-COVID-19 thyroid complications [41–48]. None of the articles discusses simultaneously COVID-19-mediated thyroid dysfunction and its phytochemical-based remediation. The present review comprehensively deals with various aspects relevant to COVID-19-induced thyroid-related complications along with the mechanism and the future outcomes in terms of treatment. Also provides perspectives on the efficacious role of phytochemicals in the treatment of COVID-19-induced thyroid disorders. Herein, we have elaborated on the mechanism of organ function dysregulation amidst COVID-19 infection along with COVID-19-mediated hormonal disbalance and its consequence. Further, the role of several phytochemicals in the management of COVID-19-associated hormonal disbalance and thyroid dysfunction has been elaborated. At last, challenges associated with the use of phytochemicals-based formulations in the management of COVID-19 have been discussed. The content of this review provides insight into the mechanism by which COVID-19 and its associated complication affect organ function of the body along with the mechanistic insight into how phytochemicals could help to cure post-COVID-19 complications in thyroid patients.

## Mechanism of dysregulation of various organ functions by SARS-CoV-2

**Figure 1:**
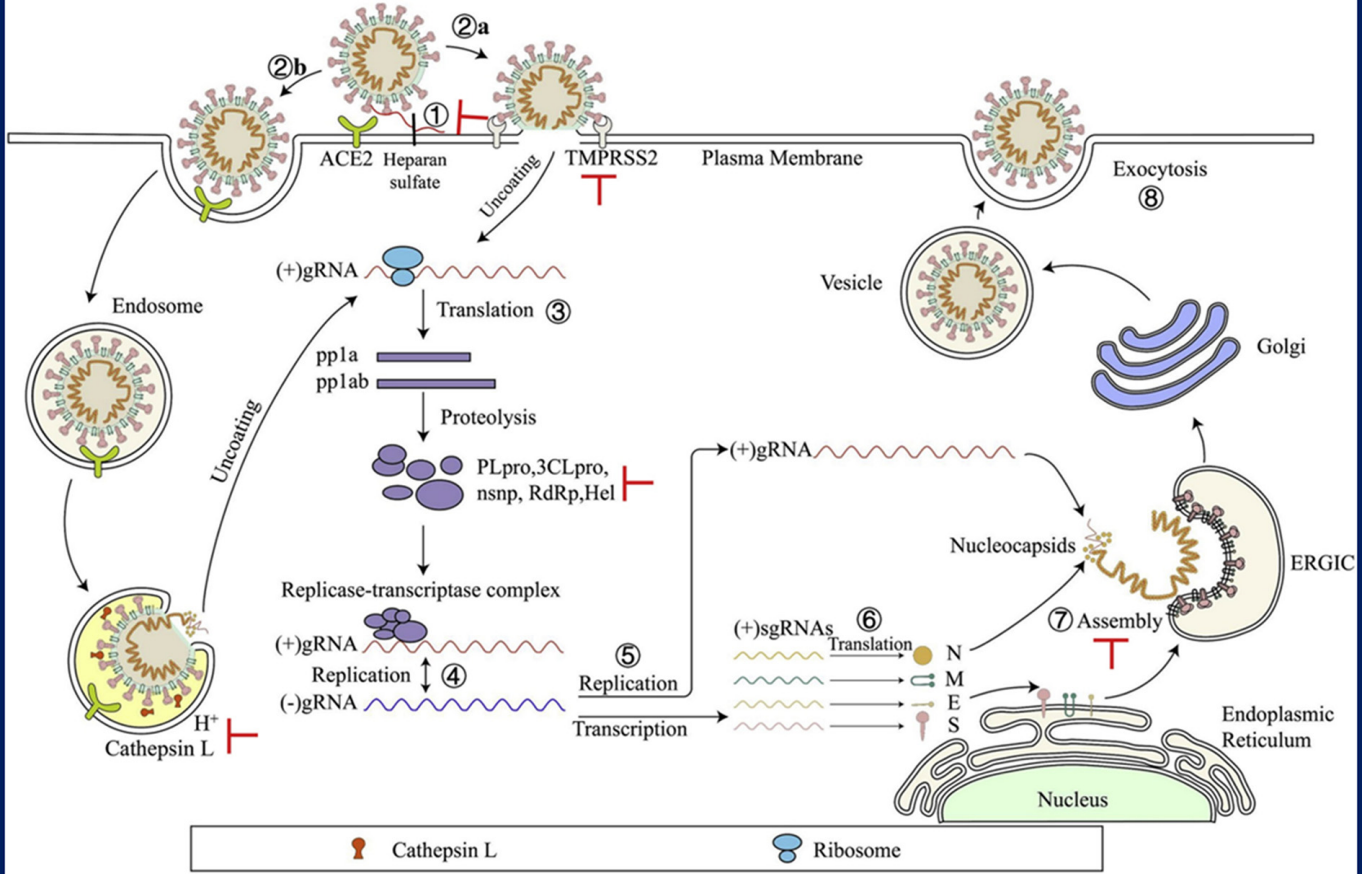
The mechanism of SARS-CoV-2 infection The virus enter via ACE-2 receptor mediated endocytosis followed by genome replication, assembly, and release of SARS-CoV-2 via exocytosis. Copyright permission from ref [ 54].

Renin, angiotensin, and aldosterone are the three hormones that make up the renin–angiotensin aldosterone system (RAAS) axis, which regulates blood pressure, sodium absorption, inflammation, and fibrosis [49]. Many disorders, including heart failure, hypotension, diabetes, and atherosclerosis, may be caused or treated by RAAS imbalances or alterations [50]. The RAS enzyme ACE2 is present on the cell surface of alveolar epithelial type 2 cells located in the lung and similar cells of various tissues of the body [280]. It serves as a receptor for the binding of SARS-CoV-2 spike protein. The binding allows the entry of viruses inside the cell. The binding affinity of ACE-2 for the spike protein of SARS-CoV-2 is 10–20 times stronger than that of SARS-CoV, which justifies pretty well its increased transmittance. The binding of spike protein to ACE2 and proteolytic cleavage by TMPRSS2 facilitate the entry of virus, replication, and cell-to-cell dissemination [51]. Moreover, SARS-CoV-2 can also get into cells through endocytosis, which is independent of TMPRSS2. In the endocytosis pathway, cathepsin L plays a major role in cleaving viral spike protein [52,53]. The mechanism of entry of the virus inside the cell has been illustrated in Figure 1.

**Figure 2:**
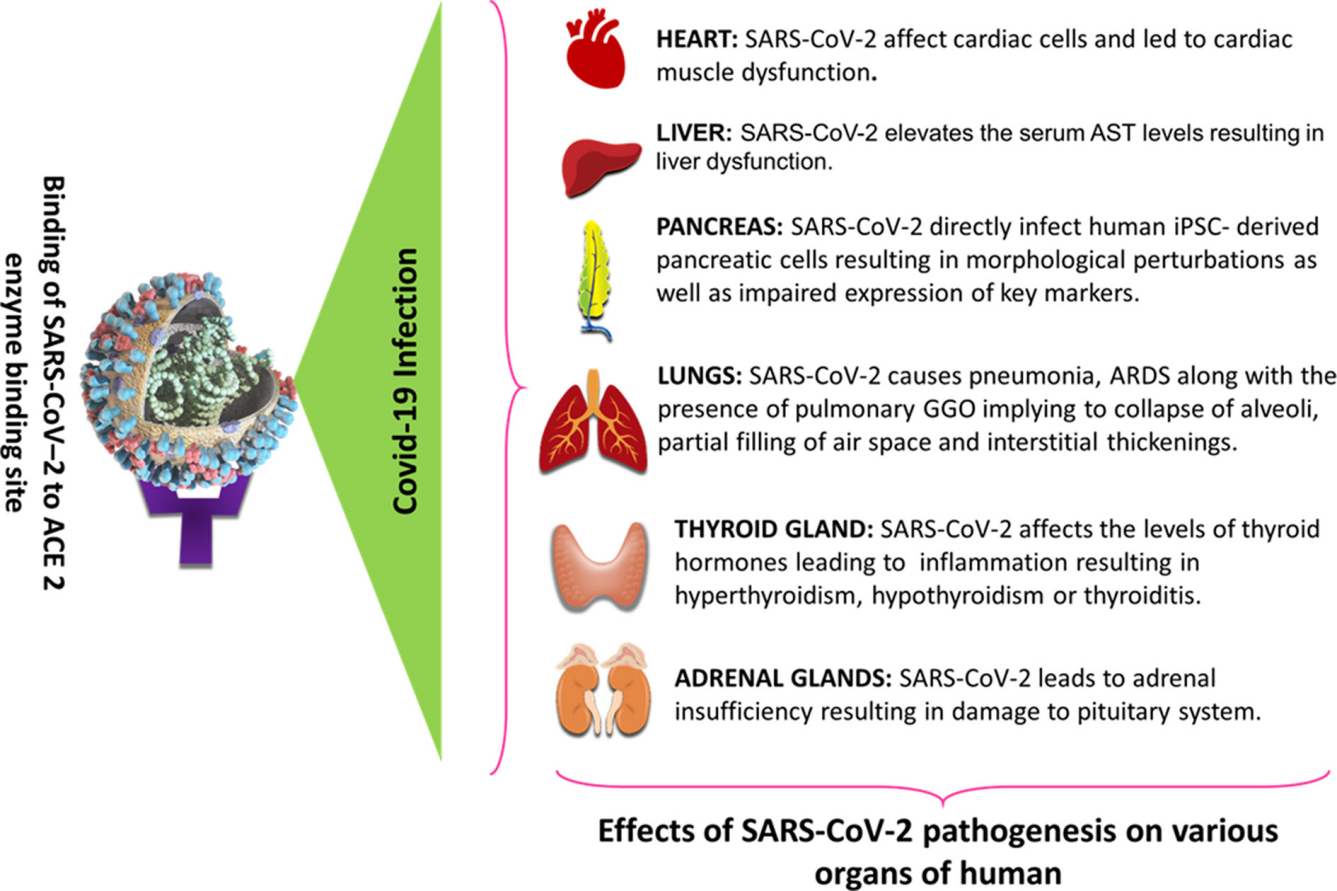
Effect of SARS-CoV-2 Infection over different body organ ACE-2 receptor mediated SARS-CoV-2 infection in various organs that led to multiple organ failure.

The ACE2 level is higher in the disease state, which could be due to a lack of response of the hyperactive RAAS activity to the compensatory response. The incidence of pulmonary edema and cough is higher in COVID-19, in part because bradykinin degradation is weakened due to lower ACE activity. SCoV’s RAAS-hyperactive axis may be regulated in part by angiotensin receptor blockers (ARB). During the infection process, SARS-CoV lowers the expression of ACE2 on the surface. In a vicious loop, reduced ACE2 activity leads to an increase in Ang II and an increase in ACE2 down-regulation, resulting in acute lung damage. In contrast, ACE2 is the main entry site, other receptors may also play a crucial role in SARS-CoV infection [55]. Due to the presence of the ACE2 receptor on various organs such as the lungs, liver, pancreas, thyroid, and adrenal gland SARS-CoV-2 infection causes damage to these organs which is depicted in Figure 2.

SARS-CoV-2 can infect the heart muscle as the cardiac cells express ACE2. Although, the viral DNA has been amplified in cardiac tissue from SARS patients who died, the idea that SARS-CoV-2 causes myocarditis remains debatable. However, it could lead to arrhythmia condition due to systemic inflammation and metabolic imbalance in some people. The level of D-dimer, a fibrin degradation result of thrombus formation, was also significantly higher in COVID-19 patients [56]. Although the increase in this marker appears to be an important predictor of death from COVID-19, the rise in the incidence of the acute coronary syndrome is related to the enhancement of these factors. Because ACE and ACE2 are two distinct enzymes, [57] ACE inhibitors have no effect on ACE2. Although it has been suggested that angiotensin II receptor blockers be used to increase ACE 2, the evidence is mixed, and each type 1 angiotensin II receptor blocker is distinct for each organ. There is no evidence to support the use of inhibitors of ACE type 1 or type II receptor blockers to facilitate coronavirus entry by increasing ACE 2 expression. The expression of ACE 2, lung injury, and inflammatory response was observed to be reduced in the lipopolysaccharide-induced acute lung injury mouse model. Injection of ACE2-transfected cells, on the other hand, can help to improve lung function and reduce lung damage. The pneumonia caused by lipopolysaccharide (LPS) was significantly reduced when these mice were given ACE inhibitors and ARB [58]. Previous research has revealed that SARS-CoV S protein can exacerbate acute lung failure by disrupting RAS regulation [59,60].

## COVID-19-mediated hormonal imbalance

Many people with severe COVID-19 appear to be extremely hypercoagulable, resulting in venous thrombosis, diffuse intravascular coagulation (DIC), and pulmonary emboli [61,62]. Moreover, amplified aldosterone release facilitated by Ang II/AT1 could be linked to thrombotic events [63]. AngII and aldosterone have been shown to boost the level of plasminogen activator inhibitor-1 (PAI-1) expression, which is found to be a key blocker of fibrinolysis *in vivo*, in endothelial cells, and in vascular smooth muscle cells [64]. The increased level of the protein C receptor on vascular endothelium by aldosterone released in response to AngII/AT1 activation is linked to the prethrombotic condition [65,66].

The generation of steroids is one of the key tasks of the ovaries and testes. As a result, measuring sex hormone levels can be used to assess COVID-19 patients’ gonadal function. Because serum testosterone (T) concentration is not frequently evaluated in clinical practice, the gonadal function of critically unwell in men but its cause is still unknown [67]. COVID-19’s effect on male reproductive hormones was investigated in recent studies. In one study, the levels of sex-related hormones in 119 SARS-CoV-2-infected men of reproductive age to 273 age-matched controls were evaluated. The majority of individuals have an illness that was found to be severe. An increased level of serum luteinizing hormone (LH) along with a lower ratio of T to LH were found in the SARS-CoV-2 infection [68]. In another study, Rastrelli et al. examined T and LH levels in male SARS-CoV-2 patients. A steady decrease in T levels and an upsurge in LH levels accompany the deterioration of the clinical state [69].

However, because these patients were unable to acquire basic linear hormones prior to infection, these findings should be interpreted with caution. Hypogonadism is also a typical symptom of systemic disease. In the case of SARS-CoV-2 infection, due to the non-specific effects of severe systemic illnesses, it is still unclear how does the reported low T levels can directly affect gonadal function as a result of COVID-19 disease [70,71]. To determine the longevity of these effects following rehabilitation, patients must be monitored and analyzed for their reproductive function. The examination of putative mechanisms should also be considered as a focus for future research [68]. Moreover, severe acute disorders in women can alter the function of the hypothalamus–pituitary–gonad (HPG) axis, lowering endogenous estrogen and progesterone levels [72].

The development of a novel coronavirus infection appears to be linked to Type 2 diabetes. In fact, the commonly occurring comorbidities caused by other coronavirus illnesses, such as in the case of SARS and MERS, have been identified as DM2 and hypertension. Affected patients with DM2 and other metabolic syndrome have shown 10 times more mortality rate and died from COVID-19, according to multiple investigations, including one from the CDC (Centers for Disease Control and Prevention coronavirus report). Although diabetes mellitus Type 2 and other metabolic diseases increase the mortality rate and associated comorbidities in many infectious diseases, however, there are other mechanistic aspects also of coronavirus infection that must be considered separately which will improve the treatment of severely infected patients. In patients with diabetes, the diagnosis of hyperglycemia and DM2 are independent predictors of death and morbidity. The fact that these patients are experiencing metabolic inflammation, which releases more cytokines might explain the mechanism that lies behind this enhanced mortality rate. Multiple organ failure in the case of COVID-19 severe illnesses is linked to cytokine storms which are basically increased levels of inflammatory cytokines. The metabolic inflammation can also harm the patient's immune system, limiting the body’s ability to fight infections, slow healing, and lengthening of recovery times. However, animal models show that co-infected DM2 can create immunological problems and lessen the severity of MERS-CoV infection. After infection, diabetic mice in which human DPP4 was expressed, led to an increase in MERS-CoV vulnerability. Mice also had an altered cytokine profile and elevated IL-17 expression. Hence, these findings confirm the theory that the combination of coronavirus infection with DM2 causes an immune response dysregulation, resulting in lung disease progression and extension [73].

Moreover, the outbreak of COVID-19 is known to cause visual impairment in about 12 subjects in accordance with a few studies. In a study by Selvaraj V et al., a middle-aged woman with SARS-CoV-2 infection struck unexpectedly and had impaired vision in her right eye. The MRI scans of the brain and eyes were both found to be normal. According to the investigation, posterior ischemic optic neuropathy was reported to be responsible for it. Apart from this, thromboembolic events, systemic inflammation linked to COVID-19, and CoV invasion via blood or direct central nervous system (CNS) invasion along with the cribriform plate and conjunctiva could also be considered as probable causes for this [74].

## Phytochemicals as therapeutic intervention for COVID-19

Phytochemical is basically referred to plant-based (phyto) chemicals, which include a varied range of substances found naturally in plants. Plant compounds of various structures and functions are referred to as phytochemicals. Certain phytochemicals in plants exhibit specific color and odor, which plays a crucial role in the protection and attraction of insects for plant pollination. Besides, they also offered defense systems to plants for instance secrete phytoalexins for pathogen defense, toxins for insect protection, allelochemicals for herbivory defense, hormones for growth and signaling, antifeedants against gazing, etc. [75,76] When ingested by humans, phytochemicals exhibit biological activity. Fruits, whole grains, vegetables, nuts and seeds, and other types of plant food products are the most prevalent sources of phytochemicals. Many phytochemicals contained in plants have been related to lowering the risk of chronic non-communicable diseases like autoimmune disorders, cardiovascular disease, and Type 2 diabetes [77]. Millions of people have been sick and perished worldwide since the outbreak of COVID-19 but so far only a few specific medicinal drugs which include remdesivir, molnupiravir, and Paxlovid have been clinically approved to treat this condition, necessitating the development and production of more innovative therapeutic agents. Lung injury and enhanced inflammation are one of the COVID-19 consequences that have gained a lot of attention.

**Figure 3:**
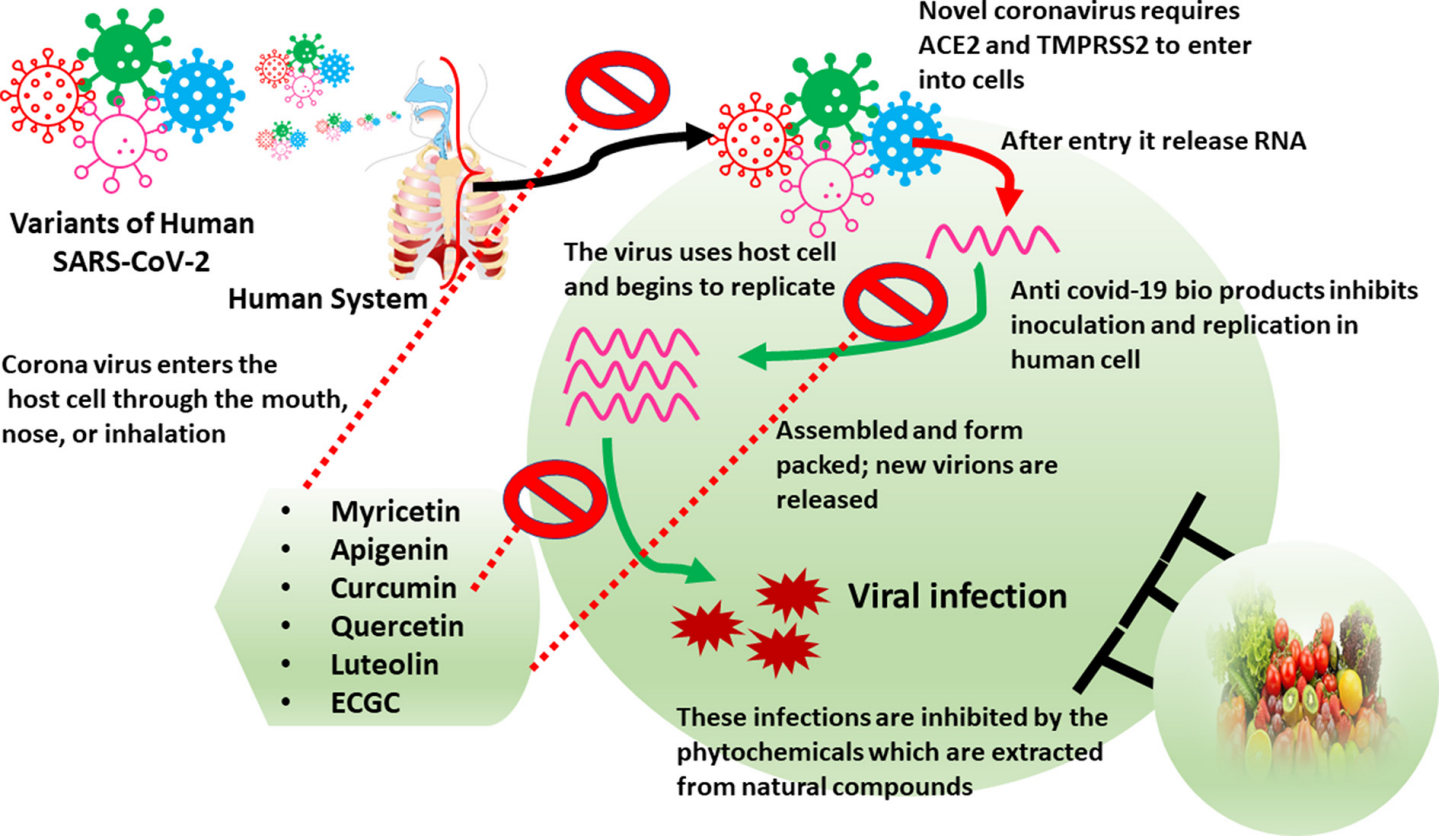
Antiviral efficacy of potential phytochemicals to inhibit SARS-CoV-2 infection The phytochemicals myricetin, apigenin, curcumin, quercetin, luteolin, and ECGC potentially inhibit the various steps of infection ranging from entry of viral particle via ACE2 and TMPRSS2 to the assembly, replication, and release from the infected cells.

The phytochemicals have been proven to exhibit powerful anti-inflammatory activities, making them useful in lowering lung harm produced by SARS-CoV-2 [46,78]. Based on their therapeutic performance five phytochemicals namely curcumin, sulforaphane, garlic extract, ginseng, and green tea extract were recently selected and subjected to clinical trials against SARS-CoV-2, influenza, or respiratory viruses. An oral nanocurcumin formulation that is registered for a clinical trial in Iran (IRC: 1228225765) is found to be efficacious against three separate COVID-19 characteristics and the findings were overwhelmingly good. In addition to volatile oils, proteins, carbohydrates, and resins, the three carotenoids curcumin, demethoxycurcumin (DMC), and bisdemethoxycurcumin (BDMC) are found in turmeric. For this to be established as a reliable medication against SARS-CoV-2, additional testing on a broad cohort is required. On minimally afflicted and asymptomatic patients, a clinical trial of ashwagandha in conjunction with Swasari Ras, Tulsi Ghanvati, Giloy Ghanvati, and Anu Taila was conducted (Clinical Trial Registry-India (CTRI); CTRI No. CTRI/2020/05/025273). It was discovered in the trial that the treatment with there has been reported to shorten the time needed for health restoration and decreased the serum levels of the pro-inflammatory markers hs-CRP, interleukin-6 (IL-6), and tumour necrosis factor-α (TNF-α) [79,80]. The antiviral efficacy of phytochemicals to inhibit SARS-CoV-2 infection has been depicted in Figure 3.

The intricate pathophysiological mechanisms behind viral illnesses as well as the adverse effects associated with current conventional medications need the introduction of newer and safer treatments. The SARS-CoV, MERS-CoV, and the other newest human coronaviruses (HCoVs) are linked to the epidemic of coronavirus illness COVID-19 as they all produce acute respiratory distress syndrome [81]. Phytochemicals could be beneficial agents in the treatment or management of various disorders due to their therapeutic effects in targeting multiple dysregulated mediators involved in disease progression [82]. TNF-α, mitogen-activated protein kinase (MAPK), interleukin-1 (IL-1), IL-8, matrix metalloproteinases (MMPs), IL-6, nuclear factor-κB (NF-κB), cyclooxygenase-2 (COX-2), reactive oxygen species, and inducible nitric oxide synthase (iNOS) are among the proinflammatory, and oxidative mediators targeted by medicinal plants-based phytochemicals. Due to the proven efficacy of phytochemical-based formulations in reducing inflammation and oxidative stress-mediated lung injury, they gained a lot of attention in the fight against coronaviruses and related fetal consequences [83,84]. Some of the phytochemicals which showed an avid role in the treatment of COVID-19 along with their respective mechanism of viral inhibition have been listed in Table 1. Some phytochemicals such as Bacalein and dieckol are not very specific inhibitors of the main protease. In this context, conflicting results were reported for their potency in inhibiting the main protease which could happen due to differences in in-vitro experimental conditions or other variable factors. The target engagement of these phytochemicals in inhibiting the main protease needs further validation in the cell culture assay and animal models. The antiviral activities of these phytochemicals might involve other mechanisms [85,86].

**Table 1:** Phytochemicals, sources, structure, and mechanism of viral inhibition

Name	Sources	Mechanism	Ref.
Epigallocatechin gallate	Green tea	Interacts with catalytic residues of main protease (M^pro^)	[87,88]
1,3,5-Trihydroxybenzene	Brown algae *Sergassumspinuligerum*	Inhibits main protease (M^pro^)	[89]
8,8′-Bieckol, 6,6′-Bieckol, Dieckol	Brown algae *Ecklonia cava*	Inhibits main protease (M^pro^)	
Quercetin	Red (grape) wines, Leaves of radish (*Raphanus raphanistrum subsp. sativus*) fennel (*Foeniculum vulgare*), and seeds of pepper (*Capsicum annuum*)	Inhibits 3CL^pro^ and PL^pro^ by binding with the proteases, Reduced RNA and Protein synthesis, and Antioxidant activity.	[90]
Epicatechingallate	Green tea	Inhibits main protease (3CL ^pro^)	[91]
gallocatechin-3-gallate	Green tea	Inhibits main protease (M^pro^)	
biflavone amentoflavone	*Ginkgo biloba* and *Hypericum perforatum*	spike (S) protein of SARS-CoV-2 binding to human ACE2 receptors via membrane fusion mechanism	[92,93]
Luteolin	*Galla chinensis*	Binds with the surface protein	[94]
Glycyrrhizin	*Glycyrrhiza glabra*	Binds with the active sites of the main protease	[95,96]
Indirubin	*Indigo Naturalis*	Binds with the main protease and hence, inhibits the viral mechanism of action	[97]
Myricetin	*Galla chinensis*	Binds with the surface protein	[98]
Baicalin	*Scutellaria baicalensis*	Blocks the entry of the virus by binding with the ACE 2 receptor, inhibits SARS-CoV-2 3CL^pro^	[99,100]
Curcumin	*Curcuma longa, Curcuma xanthorriza*	Interfering Viral replication machinery or inhibition of cellular signaling pathways crucial for Viral replication	[101,102]
Kaempferol	*Rubus idaeus, Brassica Capparis spinosa, oleracea, Phaseolus vulgaris*	Inhibit 3a ion channel of Coronavirus	[97]
Apigenin	*Matricariarecutita, Chamaemelum nobile*	Inhibit main protease and pathogenesis of SARS-CoV-2	[103]

**Figure 4:**
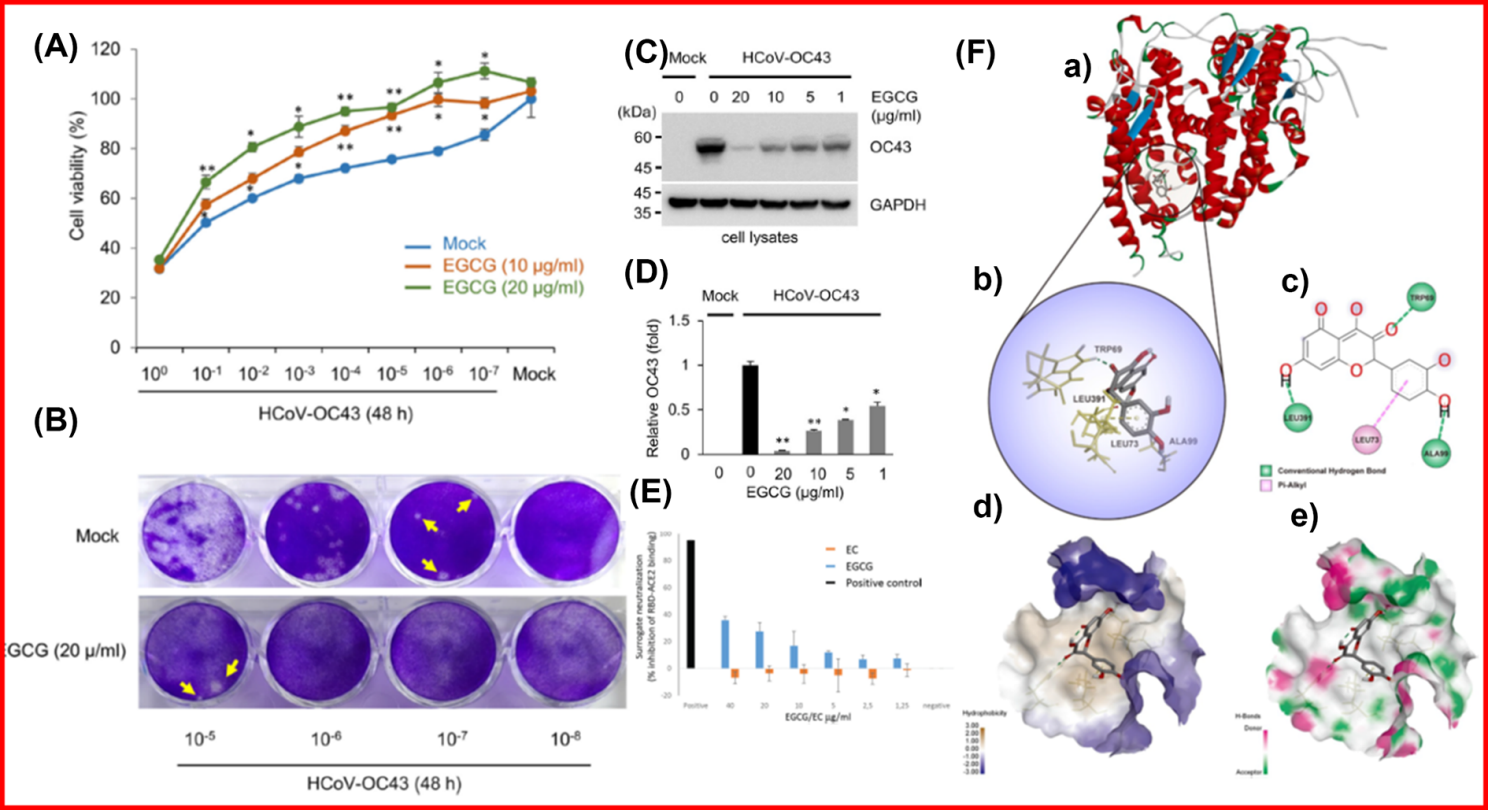
EGCG decreases the cytotoxicity caused by HCoV-OC43 virus (**A**) The RD cell was infected with mock and the HCoV-OC43 virus and an MTT assay was performed to assess cell viability. (**B**) Plaque formation was assessed by crystal violet staining. (**C**) The EGCG treatment decreases OC43 protein expression in RD cells as assessed by western blot (**D**) EGCG inhibits coronavirus in a dose-dependent manner. Copyright permission from [106]. (**E**) The sVNT assay performed with the different concentrations of EGCG showed that EGCG inhibits the attachment of SARS-CoV-2 RBD to ACE2 in a dose-dependent manner. Copyright permission from [107]. (**F**) Quercetin interaction with SARS-CoV-2 using network pharmacology and in-silico study. (Fa) Crystal structure of ACE2 (1R42) (B) Quercetin as the ligand in the active binding site. The interacting residues are shown as yellow dashed lines. (C) 2D docking pose of Quercetin with amino acids LEU391, TRP69, ALA99, and LEU73of 1R42. (D) the hydrophobic surface showing Quercetin interacts with 1R42 (E) the hydrogen bond donor-acceptor residues showed Quercetin binding with 1R42. Copyright permission from [108].

An enzyme, protease is encoded by the viral genome and is involved in the synthesis of viral proteins as well as modulating the replicase complex activity. The SARS-CoV proteins and enzymes are one of the most potential targets for the identification of anti-SARS medicines due to their critical involvement in the life cycle of the virus. The viral reproduction and infection are mediated by enzymes, making it an ideal target for antiviral drug development. Phytochemical-based antiviral medicines act as inhibitors such as betulinic acid, aloeemodine, indigo, quinomethyl triterpenoids, luteolin, quercitin, and gallates showed efficacious effects in many studies [104,105]. In this context, a study conducted by Jang et al reported that EGCG inhibits the human coronavirus and it reduces HCoV-OC43-induced cell cytotoxicity. Herein, treatment with EGCG decreases the cytotoxicity of HCoV-OC43 infections Figure 4A. The infection of RD cells with mock and HCoV-OC43 virus followed by MTT assay to assess cell viability. Further, plaque formation was assessed by crystal violet staining, and the results are shown in Figure 4B. The EGCG treatment decreases OC43 protein expression in RD cells as assessed by Western blot (Figure 4C) and EGCG inhibits coronavirus in a dose-dependent manner (Figure 4D) [106]. In another study, the sVNT assay was performed with the different concentrations of EGCG showed that EGCG inhibits the attachment of SARS-CoV-2 RBD to ACE2 in a dose-dependent manner (Figure 4E) [107]. Gu et al. assessed the efficacy of Quercetin against COVID-19-mediated acute kidney injury in an *in silico* study using a network pharmacology approach. The interaction of Quercetin with the ACE2 (PDB ID:1R42) in the active site of protein has been shown in Figure 4F. The 2D docking interactions of Quercetin revealed its interaction with amino acids LEU391, LEU73, TRP69, and ALA99 of 1R42 [108].

**Figure 5:**
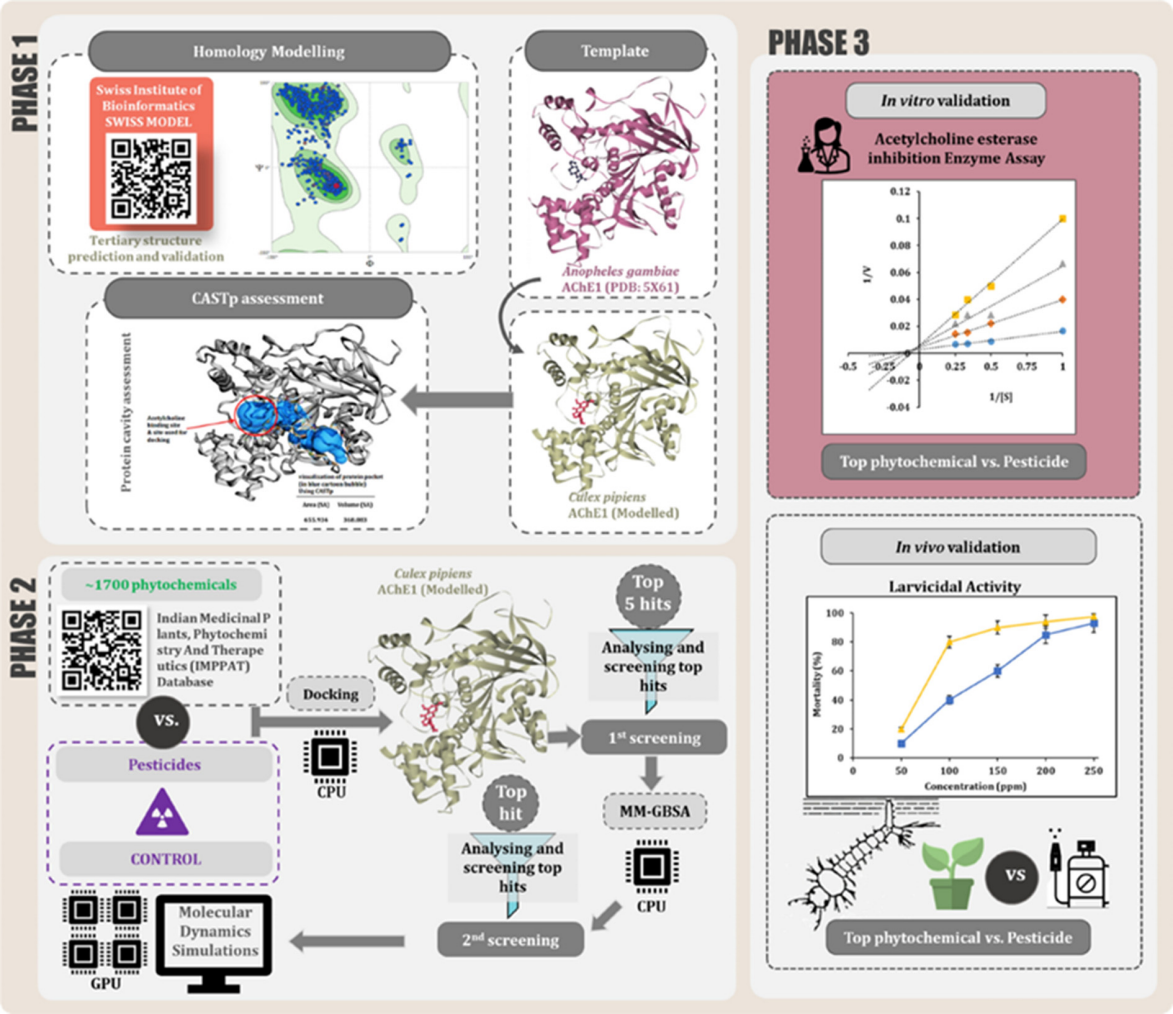
Overview of work performed in three phases In phase 1 structure prediction of Cx. pipiens AChE1 protein using an *in silico* approach. Phase 2 identifies the phytochemical that can bind with strong affinity at the catalytic site of the protein and inhibit its function. Phase 3, *in vivo* and *in vitro* bioassay to evaluate efficacy (**A**) *in vitro* AChE inhibition activity by pyridostigmine, malathion, and curcumin (**B**) Dose–response curve for malathion and curcumin. Copyright permission from [110].

The natural compounds have IC50 values ranging from 3 to 300 M, according to enzyme assays. In the early stages, SARS-CoV 3CLpro was used in the research. In a study, phytochemicals such as diterpenoids, and biflavonoids were identified from plants having potential inhibiting action of 3CLpro. Herein, ethanol extracts of phytomedicine were used. The 3CLpro active site amino acid residue and the 3-OH galooyl group were found to be essential for antiviral activity. The biologically active chemicals such as epigallocatechin gallate, quercetin, and Gallo catechin gallate (GCG) demonstrated good inhibition characteristics [109]. Quercetin is a polyhydroxy-flavonoid molecule found in plants’ flowers, leaves, and fruit that helps to boost immunity. Gallic acid, in turn, has antibacterial qualities and inhibits carcinogenic processes. A recent systematic study conducted in 3 phases by Rao et al., demonstrated curcumin as a potential therapeutic molecule to inhibit ACE-2 receptor-mediated viral infection. In the multiphase study, the first phase involves an *in silico* structure prediction of the AChE1 protein of Cx. pipiens, as the crystallized 3D structure of AChE protein of Cx. pipiens was unavailable. While in the second phase identifying the phytochemical that can bind with high affinity at the catalytic site of the protein to inhibit its function using *in silico* study was carried out. In the third phase, *in vivo*, and *in vitro* bioassay was performed, which revealed dose-dependent inhibition carried out by curcumin and malathion. Further, *in vitro* AChE inhibition activity assessment by malathion, pyridostigmine, and curcumin showed a concentration-dependent response. Figure 5 showed a systematic overview of the workflow of this particular study [110].

The SARS-CoV-2 genome is made up of ∼30,000 nucleotides [111] which codes for 16 non-structural and four structural proteins, that are required for host cell entrance and viral replication [112]. The virus binds itself to the host cell with a spike glycoprotein (S protein) found in the outer envelope of the virus. The S protein is made up of two subunits that are responsible for cell adhesion and fusion [113]. Angiotensin-converting enzyme 2 is a cellular membrane protein that is the target of the S protein binding. SARS-CoV-2 has a substantially better ability to bind to the ACE2 membrane receptor than SARS-CoV, which possesses the same binding site [114]. The endosomal protease which is a serine protease 2 (TMPRSS2), hydrolyzes Protein S, causing membrane fusion [115]. The receptor itself, as well as the proteases that cleave spike protein, could be used as therapeutic targets [116].

SARS-CoV and MERS-CoV outbreaks in the past have supplied us with information on the antiviral potential of several phytochemicals with added health benefits. Coronavirus is an RNA genome-containing virus that encrypts a protease that is required for the generation of viral proteins as well as the control of the replicase complex’s activity. Plant-derived polyphenols have the potential to be used in functional foods and medicinal formulations and are approved for human consumption around the globe. Further, the technique of implementing such preparations is quick and scalable. The plant natural products-based phytochemical could be considered as possible alternatives to treat COVID-19. SARS-CoV-2 replication was found to be inhibited by herbal preparations from the traditional Chinese medicinal plants *Gentiana scabra, Cibotium barometz, Cassia tora, Dioscorea batatas*, and *Taxillus chinensis* [117]. Other than targeting the viral main protease, phytochemicals are also known to inhibit viral entry by targeting ACE2 or viral protein, the viral papain-like protease, helicase, RdRp, and other viral proteins. The polypharmacology of phytochemicals can be advantageous to tackle the infection efficiently as such phytochemicals may inhibit more than one target simultaneously [118,119]. The development of inhibitors for SARS-CoV-2 is very crucial for both illness treatment and recurrence prevention [117]. In view of this, phytochemicals can be an efficacious treatment option for patients dealing with COVID-19 malfunctions.

## COVID-19-mediated thyroid dysfunction

**Figure 6:**
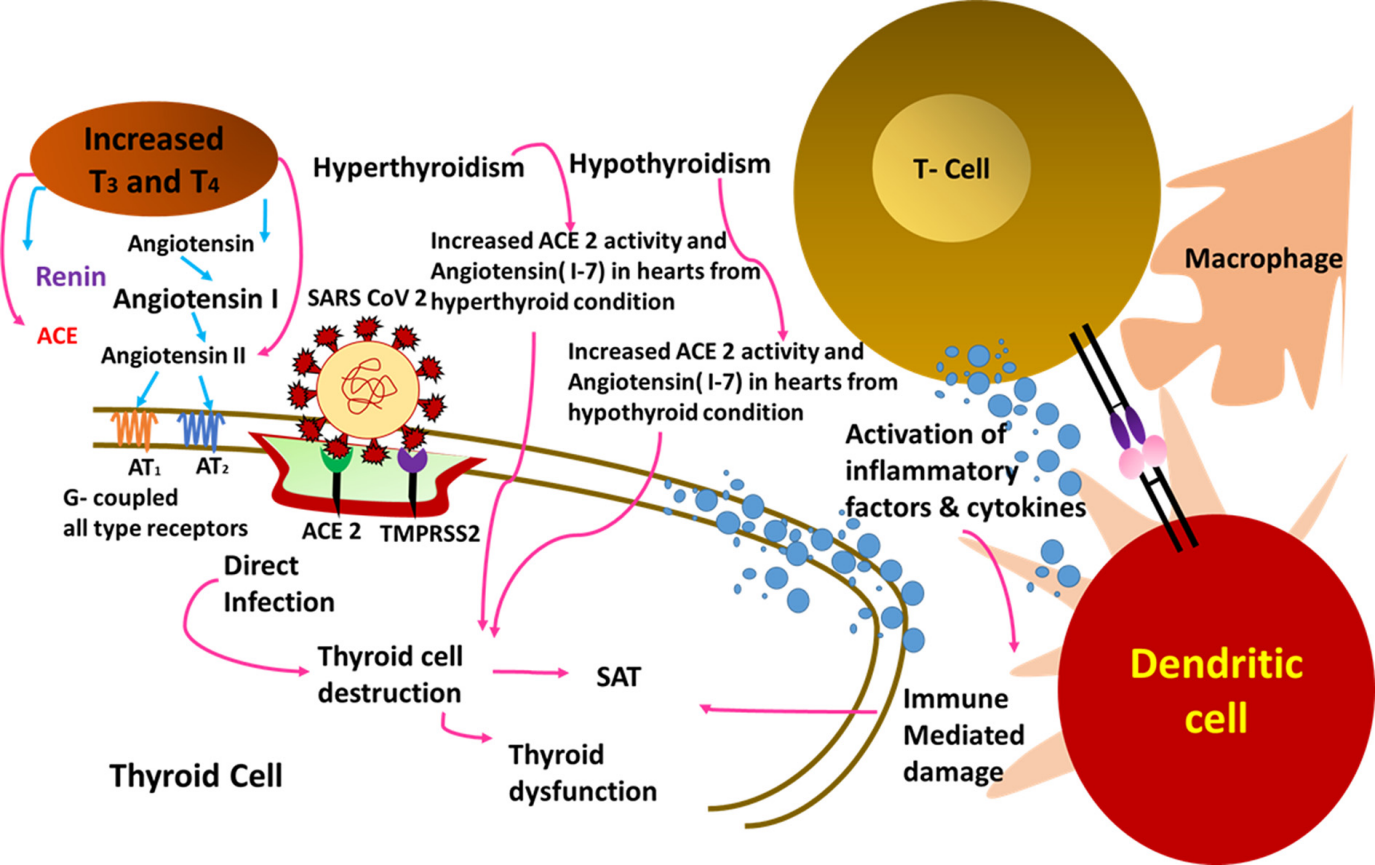
Mechanism of COVID-19 associated thyroid disorders and dysfunction The viral entry via ACE2 receptor causes its over expression that aggravate thyroid-related anomaly which include hyperthyroidism and hypothyroidism which finally resulted in thyroid cell destruction. The activation of inflammatory cytokines and immune cells led to direct damage to thyroid cells.

SARS-CoV-2 has the potential to produce pulmonary and systemic inflammation, as well as multi-organ failure. Since March 2020, the link between COVID-19 and thyroid dysfunction has been appearing at a rapid pace. The viral infection over the thyroid gland accompanied by immuno-inflammatory responses occurred in a complicated way [120, 277]. The main chemical complex used by SARS-CoV-2 to infect host cells is ACE2 coupled with the transmembrane protease serine 2 (TMPRSS2). Surprisingly, the thyroid gland has higher amounts of ACE2 and TMPRSS2 expression than the lungs [120]. Hence, the SARS-CoV-2 could have unrevealed substantial impacts on thyroid functioning. Further, the enhanced inflammatory response due to the over-activation of immune cells affects the hormone-production capability of thyroid glands. Thyroid diseases linked to COVID-19 include dysfunctions like thyrotoxicosis, hypothyroidism, nonthyroidal sickness syndrome, etc. The mechanism of thyroid dysfunction caused by SARS-CoV-2 infection has been illustrated in Figure 6.

In an investigation, 191 patients suffering from COVID-19 were enrolled into consideration among which the majority of the individuals had euthyroidism. They were reported with moderate TSH and FTH decreases, with a consistent non-thyroidal disease condition. According to the investigators, the thyroid function tests of the survivors amongst these were returned to baseline during follow-up [121]. Another research involving 191 patients with COVID-19 was undertaken in which there were 84.3 percent of patients were mild, 12.6 percent were moderate, and 3.1 percent were severe with the disease. Among these 13.1 percent of the people had thyroid dysfunction [122]. Further, it was observed that as the severity of COVID-19 increases, the FTH decreases. Those patients who are with a low FTH have a greater risk of COVID-19-associated complications. This study concluded that SARS-CoV-2 infection could affect thyroid function directly which could lead to manifestations like exacerbating pre-existing autoimmune thyroid illness. Low FTP levels linked to systemic inflammation may also have prognostic implications [123].

Furthermore, retrospective research was conducted on 76 patients, with 48 patients testing positive for COVID-19 and the remaining 28 patients having negative polymerase chain reaction (PCR) tests. In another report on HRCT, thyroid functions, IL-6, and procalcitonin were used to differentiate between moderate, severe, and serious pneumonia. They also found that 75 percent of COVID-19-positive patients had thyroid problems and had higher IL-6 levels. A logistic regression analysis also reveals that TT3, IL-6, and procalcitonin could all be risk factors for coronavirus on their own. They reported that IL-6 could be the most sensitive marker, and TT3 and procalcitonin could be predictors for COVID-19 disease, based on ROC curve analysis. As per local COVID-19 protocol, 26 patients (43.3%) in the mild group, 16 patients (26.7%) in the intermediate group, and 18 patients (30%) in the severe group were categorized in a study of 60 patients with COVID-19. The thyroid hormone assays, including total T3, total T4, free T3, TSH, free T4, and anti-TPO antibodies, as well as other baseline investigations, were used to evaluate these patients. Thirty-five percent of these patients were also found to have one or more abnormalities in thyroid function, the most prevalent of which was low TSH. Thyroiditis was diagnosed in 18.33 percent and 9.1 percent of the patients, respectively [124]. Another study discovered a prognostic effect of thyroid disorders over the severity of COVID-19 clinical cases. They examined at how thyroid disorders and thyroid gland nodules affected the prognosis of 125 patients with COVID-19. These patients were evaluated and divided into two groups: first and second. Patients in the first group had mild symptoms in the non-ICU, whereas patients in Group 2 were in critical condition in the intensive care unit (ICU). These ICU patients were further separated into two subgroups: survivors (*n*=88) and deceased (*n*=37) [125].

In another case study, researchers diagnosed a 37-year-old healthy female with odynophagia and anosmia having no other respiratory illness. She suffered from COVID-19 and was prescribed symptomatic treatment for her mild condition. Following treatment, she completely recovered within a few days. After a month, she visits an ENT doctor because she was experiencing acute neck discomfort that was spreading to her right jaw and ears, as well as exhaustion. Even though she had no clinical indications of hyperthyroidism, she was referred to an endocrinologist with the doubt of SAT. Her physical examinations at the time revealed just a mildly enlarged painful thyroid gland and neck adenopathies. Further, her lab tests revealed a high ESR (72 mm/h) and CRP (66 mg/L), as well as anemia (Hb: 10.4 g/dL) and normal platelet and leukocyte counts. Besides, the thyroid testing revealed that she had hyperthyroidism with avery low TSH level. However, TSH, T4 total 13.5 mcg/dL, T4 free 1.6 ng/dL, and T3 total of 21.1 ng/dL were all found to be normal. The anti-Tg and anti-TPO antibodies were both negative. There was no radioactive iodine uptake on a thyroid iodine scan. Hence, the subacute thyroiditis diagnosis was confirmed and seems to be due to post-COVID-19 complications [126–129]. Muller et al. conducted a study concentrating on the SAT prevalence and thyroxine thyrotoxicosis in some patients with severe COVID-19 admitted to the ICU unit [130].

The time between a COVID-19 diagnosis and the onset of symptoms was anywhere between 5 and 1 month, but the time between an SAT diagnosis and recovery was found to be between 1 week and 1 month. In contrast with Muller et al. findings, which looked at thyroid function tests in ICU patients in 2019, without COVID-19, and ICU patients in 2020, with COVID-19. In 2020, a higher proportion of ICU patients had increased TSH, indicating the SAT associated with COVID-19. Seven individuals with low TSH levels were monitored for 55 days to examine if their symptoms were consistent with SAT. Only three of the patients had imaging scans that were compatible with SAT, despite having laboratory values associated with SAT without ever feeling neck pain. According to these findings, SARS-CoV-2-mediated thyroid dysfunction might manifest with or without clinical symptoms.

A female was diagnosed with pneumonia five days after being diagnosed with COVID-19, according to Ippolito et al., palpitations, sleeplessness, and anxiousness were the main symptoms [131]. The neck problems were not reported, but she was on pain medication. Her TSH was low, her FT3 was high, and she tested negative for anti-peroxidase antibodies (TPOAb), anti-thyroglobulin antibodies (TgAb), and anti-TSH receptor antibodies (TRAb), [131] Following 15 days after a COVID-19 positive oropharyngeal swab with undetectable TSH, and increased FT4 and FT3. Brancetella et al. described a female with fever, neck ache radiating to jaw and palpitations. Increased levels of TPOAb, TgAb, TRAb, white blood cell count, and inflammatory markers were also observed in this case [131]. One month after being diagnosed with COVID-19, the patient had neck pain, fatigue, tremors, and palpitations, according to Ruggeri et al. TSH was repressed, FT4 and FT3 were increased, and TgAb, TPOAb, and TRAb were undetectable in the patient [132]. A female patient with acute neck discomfort (8/10) extending to the right jaw and ear, following exhaustion, was described by Campos-Barrera et al. She made no mention of any hyperthyroidism symptoms. Her TSH was undetectable, but her FT4 and FT3 levels were high. TgAb, TPOAb, and TRAb were undetectable, along with observed anemia [133].

When compared with control groups, mean TSH readings in COVID-19 patients were considerably lower [134,135]. The lower TSH amount was observed abnormally low in 15–56% of patients with COVID-19 in conjunction with a varied range of FT3 or FT4 ranges from low or normal to high; however, high TSH levels were recorded in ∼ 8% of patients with COVID-19, as reported by several recent studies [133,136–142]. Thyroid dysfunction was observed to be considerably more common in COVID-19 patients in these investigations which include mainly healthy controls with or without COVID-19 ARDS. Several investigations indicated a link between thyroid malfunction and COVID-19 clinical severity, with a decline in TSH and FT3 amount which shows a strong positive correlation with illness severity [143–145]. In a study conducted by Chen et al., cases with COVID-19 were graded clinically as severe, moderate, or critical and the severity of the condition was positively linked with the amount of TSH and total T3 (TT3) reductions [144]. Total T4 (TT4) levels, on the other hand, were not linked to the severity of sickness. TSH levels were found to be lower in severe and critical COVID-19 cases when compared with non-COVID-19 pneumonia cases of similar severity [143]. The FT3 levels, TSH, and the FT3/FT4 ratio were considerably low in severe cases of COVID-19 patients than in non-severely affected patients, according to Gao et al [144]. While the majority of the patients' TSH readings were still within normal limits in the non-severe patients’ group. There were no variations in FT4 levels recorded [144]. Lui et al. found similar findings, stating that the decline of FT3 levels linked with systemic inflammation appeared to be connected to the clinical worsening of symptoms in patients, although TSH and FT4 levels were not significantly affected [123]. Zou et al. found significantly low FT3 levels in 27.5 percent of COVID-19 patients; these were older females patients with more severe symptoms including fever and dyspnea [146]. The low TT3 or FT3 levels associated with thyroid dysfunction are common in severe clinical situations and are referred to as non-thyroidal illness syndrome (NTI) or ESS [123].

In a special cohort of 287 COVID-19 patients, 20.2% of patients were reported to have thyrotoxicosis, having TSH levels below the normal range, and multivariate analysis revealed an opposite connection between TSH values in serum and cytokine interleukin-6 (IL-6 levels) [147]. Further, FT3 levels were also inversely correlated with C- reactive protein (hs-CRP), and TNF-α in the study carried out by Gao et al., whereases TSH levels were negatively correlated with hs-CRP and IL-6 in the entire evaluated population independent of disease severity but not in the non-survivor group [144]. Enhanced CRP levels were independently correlated with low FT3 levels in Lui et al studies as well as greater procalcitonin levels in Zou et al investigations. Gao et al. investigated whether thyroid hormone levels could predict mortality in COVID-19 patients with severe symptoms [123,144,146]. Higher FT3 levels were linked to a lower risk of all-cause mortality, according to the researchers. Further, in research by Lania et al., a 21.4 percent in-hospital death rate was reported, along with low TSH levels in 20% of patients and in nearly 40% of patients with thyrotoxicosis with TSH levels of 0.1 mU/L [145]. Moreover, patients with euthyroidism or hypothyroidism spent more time in the hospital. TSH and T3, and T4 levels were measured after recovery in two investigations, and both returned to baseline at the follow-up [148,149].

Thyroid hormone levels should be carefully considered when interpreting low TSH values. Thyroid hormone level was significantly noted in only 73 of the 287 individuals studied by Lania et al., [145] and 31 of them had thyrotoxicosis with FT4 levels higher than normal range along with normal TSH- receptor antibody (TRAb); however, no imaging results were available. In the study by Muller et al., 25% (*N*=2) of patients with low levels of TSH had hypothyroidism, noticeable hypoechogenicity, and crucial heterogeneity in ultrasound during the recovery period, but in association with a mild hypoechoic pattern at neck ultrasound, while 75% (*N*=6) of patients with low TSH levels presented with hyperthyroidism, clear hypoechogenicity, and heterogeneity as observed in ultrasound during the recovery period. During follow-up, thyroid function improved, but it was accompanied by a slight hypoechoic form on neck ultrasonography and, in some cases, a lower uptake on 99mTcpertechnetate scintigraphy. Thyroid dysfunction has more likely been linked to thyrotoxicosis caused by the subacute thyroiditis phenomena along with ESS in both trials, with antibodies (anti-Tg, anti-TSHR, and anti-TPO) being negative in the majority of circumstances.

A migrant laborer from Myanmar was reported to have had subacute thyroiditis in combination with COVID-19 in a one-of-a-kind instance. He suffered sinus tachycardia and anterior neck pain on the day of his sickness, and thyroid function testing revealed primary hyperthyroidism. His thyroid gland was ultrasonographically confirmed to have subacute thyroiditis, and oral corticosteroids were given to him, which resulted in quick recovery. A 34-year-old Myanmarese male was admitted to Singapore General Hospital’s emergency department with a 4-day history of fever, headache, dry cough, and ansomnia [150]. On admission, a 37.7°C fever, 120/90 mm Hg blood pressure, 89 beats/ minute heart rate, 19 breaths/minute respiratory rate, and 96% oxygen saturation (SpO_2_). The unusual noises on the auscultation of the lungs were not observed. An oropharyngeal swab and COVID-19 testing were performed based on the clinical characteristics and risk factors, and the results were positive for COVID-19. The patient complained of a persistent dry cough and sore throat on the third day of his admission. Paracetamol and lozenges were prescribed to relieve the symptoms. He suffered from pain at the anterior neck part with a score of 5/10, which was unresponsive to therapy. An onset of tachycardia in which heartbeat ranges from 90 to 120 beats/minute starting on the 5th day of his hospital stay (day 9 of his illness). On room air, he remained afebrile with a SpO_2_ of >96%.

A diffuse asymmetric goiter was discovered on examination of his neck, with hard and painful patches on both lobes. There was no palpable bruit or retrosternal extension and only some cervical lymph nodes on both sides were palpable [151]. No thyrotoxicosis symptoms, pretibial myxoedema, or hand tremors were observed. In addition, there was no exanthem found on his skin. Thyroid function testing revealed primary hyperthyroidism with high free T3 (13.4 pmol/L), free T4 (41.8 pmol/L), and low TSH hormone (0.01 mU/L) in the presence of tachycardia and a painful goiter. Antibodies to thyrotropin receptor (TRAb) and thyroperoxidase (TPOAb) were absent. The CRP level was also significantly increased, reaching 122 mg/L, but procalcitonin was unimpressive (0.13 g/L). Without hyperbilirubinemia, alkaline phosphatase was modestly increased (218 U/L). The sinus tachycardia rhythm was observed in ECG with no signs of atrial fibrillation.

In a recent study, the effects of mild-to-moderate COVID-19 on thyroid function in subjects without a history of thyroid disease following full recovery were monitored. In the evaluation, 2 months after the initial SARS-CoV-2 infection, the TSH, free fT4, and antithyroid antibodies in the samples of 113 patients (median age, 43 years; 31.0% male) were measured. The level of TSH and fT4 were assessed once more after one month. Two months following COVID-19, 61.1% of the patients were found to have thyroid dysfunction of which 78.3% had subclinical hypothyroidism, 13% had preclinical hyperthyroidism, and 8.7% had overt hypothyroidism. The presence of thyroglobulin antibodies, the need for levothyroxine medication, and a higher likelihood of thyroid dysfunction were all substantially linked with moderate rather than mild manifestations of COVID-19 (OR 5.33; 95% CI: 1.70–16.69, *P*=0.002). Approximately 28.3% of the individuals still had subclinical hypothyroidism at the follow-up. Also, the patient’s TSH levels were found significantly lower than they were observed in the second month following their initial COVID-19 infection (*P=*0.001), but not those with subclinical hypothyroidism or those who were not receiving hormone replacement treatment [152]. The outcome of the study suggested that COVID-19 may impair thyroid function over the long term. Thyroid function testing should therefore be incorporated into the COVID-19 survivor follow-up methodology.

In another observational study by Bagala et al., the prevalence of hypothyroidism in older COVID-19 patients was examined, and it was determined whether this comorbidity is connected to a particular pattern of symptom development and a poorer prognosis. The GeroCovid acute wards cohort of COVID-19 inpatients aged less or equal to 60 years were included in this analysis. Medical records and the administration of thyroid hormones were used by them to reconstruct the history of hypothyroidism. Sociodemographic statistics, comorbidities, disease-onset symptoms and signs, and inflammatory markers were compared between individuals with and without a history of hypothyroidism. The Cox regression-based showed a relationship between hypothyroidism and in-hospital mortality. Approximately 8.5% of the total 1245 patients had a history of hypothyroidism. Compared with patients without a history of hypothyroidism, these patients were found more likely to have obesity and arterial hypertension. Patients with hypothyroidism had less frequently low oxygen saturation and anorexia in terms of COVID-19 clinical presentation, but more frequently reported muscle discomfort and loss of smell than those without hypothyroidism. Patients with hypothyroidism showed increased lymphocyte levels among the inflammatory indicators.

Hypothyroidism was only related to decreased in-hospital mortality in the univariable model at Cox regression (HR = 0.66, 95% CI: 0.45–0.96, *P*=0.03); however, after correcting for potential confounders, no meaningful outcome was seen (HR = 0.69, 95% CI: 0.47–1.03, *P*=0.07). Further, they concluded that although it may be linked to distinctive clinical and biochemical aspects during the disease's inception, hypothyroidism does not appear to have a significant impact on the prognosis of COVID-19 in older individuals [153]. To analyze the long-term outcome of thyroid problems in individuals with severe COVID-19, another study was carried out on 183 individuals with severe COVID-19 who had no previous thyroid history (baseline). They offered patients a 12-month longitudinal follow-up that included an ultrasound, testing for autoantibodies, and thyroid function. Individuals who had US focal hypoechogenicity (focal hypoechogenicity), suggestive of thyroiditis, also had thyroid ^99m^Tc or ^123^I uptake scans.

After being excluded from the TSH analysis at the outset, 63 out of 183 (34%) COVID-19 patients had started using steroids before being admitted, and 12 (10%) of them had atypical thyroiditis. Following up with 75 patients revealed normalization of thyroid function, inflammatory markers, and no rise in the incidence of thyroid autoantibodies that could be detected. Sixty-five patients had baseline US results available, and 28% of those patients exhibited localized hypoechogenicity, with 82% of those patients having decreased thyroid 99mTc/123I uptake. Low baseline TSH (*P*=0.034), high free-thyroxine (FT4) (*P*=0.018), and high interleukin-6 (IL6) (*P*=0.016) were all linked with the existence of localized hypoechogenicity. After 6 and 12 months, 87% and 50% of patients, respectively, still had focal hypoechogenicity, though it had shrunk in size. Thyroid ^99m^Tc/^123^I uptake was reduced in 67% of patients after 9 months but partially recovered from baseline (+28%). These results suggest that COVID-19 causes modest, temporary thyroid impairment [154]. It does not appear that thyroid autoimmunity is related to focal hypoechogenicity, which may continue after a year while shrinking in size and is associated with baseline high FT4, IL6, and low TSH. However, this study needs to be validated in a larger cohort as consequences, in the long run, seem doubtful.

## Phytochemical-based formulations for the treatment of COVID-19 induced Thyroid Complication

Thyroid disease refers to a group of medical diseases marked by disruptions in thyroid functions and thyroid signaling homeostasis. Thyroid illnesses are manifested in a variety of ways, the most visible and prevalent of which include hypothyroidism, which is defined by a decrease in thyroid hormone (TH) production and/or circulation; hyperthyroidism, which is characterized by an increase in TH production and/or circulation; and numerous forms of thyroid malignancies. There are a few other anatomical anomalies of the thyroid gland that are less prominent. The emergence of these diseases has been attributed to several hereditary and environmental variables. The dietary iodine consumption is a significant predictor of thyroid dysfunction risk, whereas other factors for instance age, gender, ethnicity, genetic susceptibility, endocrine disruptors, smoking status, and novel therapeutics, such as immune system blokers, may also have an impact on thyroid disease epidemiology [141].

**Figure 7:**
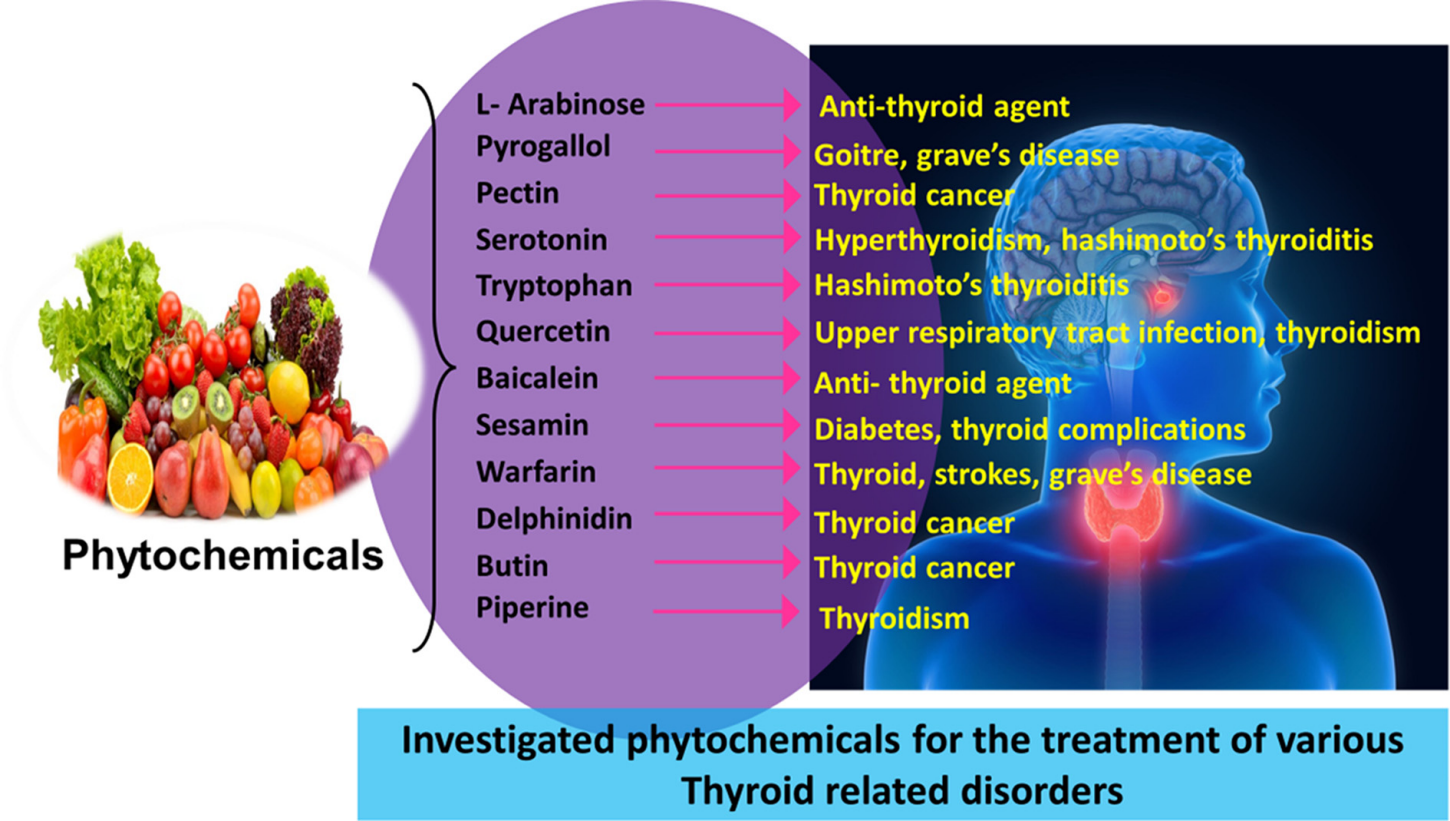
Phytochemicals which have proven efficacy for the treatment of thyroid disorders Various phytochemicals investigated for the treatment of thyroid anomaies

Thyroid function has been demonstrated to be influenced by phytochemicals in either a positive or negative way. For example, the consumption of isoflavones and flavonoids present in soybeans, such as daidzein and genistein are usually recognized as phytoestrogens, and can cause goiter and hypothyroidism [155]. This happens particularly in locations where iodine intake is inadequate. Several critical enzymes, proteins, membrane transporters, and nuclear membrane receptors play a crucial role in TH production, transport, excretion, metabolism, and nuclear receptor transactivation may be affected by flavonoids. As a result, concerns have been raised regarding the potential for some meals high in these bioactive chemicals to disturb the endocrine system, particularly the thyroid [156]. However, recent evidence suggests, that plant-derived substances such as quercetin, myricetin, apigenin, naringin, rutin, hesperidin, curcumin, and genistein can be used as adjuvants in the treatment of thyroid malignancies [157]. A schematic in Figure 7 has shown the role of phytochemicals in the treatment of various endocrine-related disorders. Table 2 shows the key roles of some phytochemicals along with the in-vitro and *in vivo* studies which shed light on the effect of phytochemicals-based formulations used for the treatment of thyroid-related disorders.

**Table 2:** Phytochemicals used for the treatment of thyroid dysfunction and thyroid-related disorders along with some phytochemicals which could play a crucial role in the treatment of COVID-19-induced thyroid dysfunctions

Phytochemicals	Sources	Thyroid Diagnoses	*In vitro* study	*In vivo* study	Clinical Trials	Diagnoses in other diseases	References
Pyrogallol	Oak, eukalyptus	Goiter, Graves’ disease, hashimoto thyroiditis, thyroid cancer	rat		Patients with Graves’ disease, Hashimoto’s thyroiditis,differentiated thyroid cancer, and endemic goiter as well as in normal thyroid tissue (paranodular tissue) from patients with follicular adenoma	Patients with follicular adenomas	[148,149]
L- Arabinose	Gum Arabic, instant coffee, wine and sake	Thyroid functioning, thyroid cancer	Rat, rat intestinal mucosa	Rat thyroid follicular epithelial cells	Patients treated with auto- and allo-haematopoietic stem cell transplantation	Auto and allohematiopoietic stem cell transplantation	[158–162]
Pectin	Pears, apples, guavas, quince, plums, gooseberries, and oranges and other citrus fruits	Thyroid cancer	Balb/C mice, rat, rats	Thyroid carcinoma cells, thyroid cancer cells		Anti- salmonella drugs	[28,163–166]
Serotonin	Eggs, cheese, pineapple, tofu, salmon, nuts and seeds	Hypothyroidism, thyroid functioning	Hippocampus, rats, rat brain,Euthyroid mice	Rat brain monoaximes oxidases, rat thyroid epithelial cell line, mouse embryo, Parafollicular cells PC) of the sheep	Hypertension, depressed patients with primary hypothyroidism or normal thyroid function	Hypertension, depression	[167–174]
Tryptophan	Milk, canned tofu, oats, cheese, nuts and seeds	Hashimoto’s thyroiditis, Graves’ disease, thyroid complications	Rat liver	HepG2 human hepatoma cells, rat erthrocytes	67 thyroid patients, 49 patients with Hashimoto's thyroiditis, 35 with Graves' disease, and 34 healthy subjects	Hepatoma cells	[175–179]
Quercetin	Citrus fruits, apple, onion, parsley, berries, green tea, and red wine	Thyroid functioning	Mice	HepG2 cells, Fisher Rat Thyroid cell Line FRTL-5 thyroid cells, thyroid type 1 iodothyronine deiodinase activity	Quercetin supplementation and upper respiratory tract infection, pharmacokinetics	Upper respiratory tract infection, pharmacokinetics, alcoholic liver diseases	[180–183]
Baicalein	Roots of *Scutellariabaicalensis* and *Scutellarialateriflora*, Olive leaves	Thyroid functioning	Rat intestinal alpha-glucosidase, female mice, breast cancer cells transplantation tumor model	PR-B and T47D cells, OVCAR5 cells, MCF-7 and MDA-MB-231 breast cancer cells, thyroid type 1 iodothyronine deiodinase activity		Breast cancer, cancer	[109,184–186]
Sesamin	Sesame seeds (*Sesamum indicum L*.)	Thyroid cancer	Thyroid cancer cell lines (FTC-133),	Adult Female Albino Rats, diabetic rats	48 patients with Type 2 diabetes	Type 2 Diabetes	[187,188]
Warfarin	sweet” clover and tonka beans	Hyperthyroidism, graves' disease			Three patients on concomitant amiodarone and warfarin, hyperthyroid patients, patient with graves' disease, patients with nonrheumatic atrial fibrillation, Warfarin-induced hypoprothrombinemia	Hypoprothrombia, non- heumatic atrial fibrillation	[189–192]
Delphinidin	pigmented vegetables and fruits, particularly blueberry	Thyroid cancer		Human CRC cell lines, human Thyroid cancer cells, HCC cells, NSCLC cell, HepG2 cells, human liver carcinoma cell line, osteosarcoma cell lines	Mice	Cancer, liver carcinoma, osteosarcoma	[29,193–196]
Butin	*Acacia mearnsii, Vernonia anthelmintica* and *Dalbergia odorifera*	Thyroid cancer		SW579 cells			[197]
Piperine	Piper nigrum fruits, *Piper longum Linn*	Thyroid functioning		Adult male Swiss albino mice, Mice, Male wistar rats			[198–202]
Apigenin	*Matricariarecutita, Chamaemelum nobile*			BCPAP cells	Mice		[203,204]
Myricetin	*Galla chinensis*			SNU-80 HATC cells			[205]
Curcumin	*Curcuma longa, Curcuma xanthorriza*			TPC1 thyroid cell line, TPC1 papillary thyroid cancer cell line			[206,207]

We have enlisted below a few phytochemicals which have a profound efficacious role in the treatment of thyroid and various thyroid-relevant disorders.

### Pyrogallol

It can be extracted from natural extracts like oak and eucalyptus. Various *in vitro* and *in vivo* trials have shown its impact on thyroid functioning and its role on various other counterparts of the human body. Pyrogallol has been found to play a vital role in the treatment of diseases like Goiter, Graves' disease, Hashimoto thyroiditis, thyroid cancer, etc [148]. Many clinical trials have also been conducted to ensure its implications in various other diseases. Some of the clinical trials on patients with Hashimoto’s thyroiditis, Graves’ disease, endemic goiter, and differentiated thyroid cancer, as well as in normal thyroid tissue from patients with follicular adenomas have been conducted. Regardless of thyroid, it has also been found to treat other diseases which may include follicular adenomas [149].

### L-Arabinose

It is a phytochemical which could be extracted from gum Arabic, instant coffee, wine, and sake. Its efficacy has been investigated in the treatment of different thyroid-relevant malfunctions as well as thyroid cancer. Many *in vitro* and *in vivo* research have been undertaken to determine the effect of L-arabinose on numerous factors [208,209]. Aside from thyroid dysfunction, many clinical investigations have also been carried out to get an insight into the role of L-arabinose on various organ systems in the human body [210–212]. Niedzielska et al., in a study evaluated selected endocrine problems in autologous and allologous hematopoietic stem cell transplant recipients which has shown L-arabinose to be effective in treating disorders such as auto and allo-hematopoietic stem cell transplantation [213]. Bronk et al have studied the influence of the accumulation of sugars on the thyroid gland in rat intestinal mucosa during absorption [214].

### Pectin

Pectin is a kind of phytochemical that could be extracted from various citrus fruits such as Pears, guavas, apples, plums, quince, oranges, gooseberries, etc. It has been found to be efficacious in the treatment of thyroid malignancies. Several *in vitro* and *in vivo* studies such as by Khotimchenko et al., on Balb/C mice and rats and a study on thyroid cancer cells by Menachem et al and Zheng et al., have reported the efficacy of pectins on various diseases relevant to thyroid dysfunction [215,216]. Pectin has not only been found to cure only thyroid-relevant aspects but Khotimchenko et al. and Stokstad et al., have investigated its effect on diseases caused by salmonella and hence, acts as an anti-salmonella drug as well [217,218].

### Serotonin

Eggs, cheese, pineapple, tofu, salmon, nuts, and seeds are major sources of phytochemicals like serotonin. Its efficacy as a cure for hypothyroidism and thyroid dysfunctioning has been well proven. Several *in vivo* studies investigations in hippocampus, rats, rat brain, and euthyroid mice have been conducted to ensure its role [219,220]. Various *in vitro* studies such as rat brain cells monoaximes oxidases, rat thyroid epithelial cell line, mouse embryo, and parafollicular cells (PC) of the sheep have also been conducted to make sure about its important role in numerous illnesses [221,222]. To corroborate its impact on other diseases regardless of thyroid, certain clinical trials have also been taken into consideration such as hypertension, depressed patients with primary hypothyroidism, or normal thyroid function. Clinical trials which ensure its treatment in not only thyroidism and thyroid-relevant dysfunction but also for the treatment of hypertension have been conducted [223].

### Tryptophan

Tryptophan whose major sources include milk, canned tofu, oats, cheese, nuts, and seeds has a great impact on the treatment of Hashimoto's thyroiditis, Graves' disease, thyroid complications, etc [224,225]. Several *in vitro* and *in vivo* studies have shown its importance in numerous illnesses which includes thyroid as a major issue. The clinical trials on 67 thyroid patients, 35 with Graves’ disease, 49 patients with Hashimoto’s thyroiditis, and 34 healthy subjects have been done in mere past to inculcate its influence on thyroid-relevant diseases [226,227].

### Quercetin

Quercetin which has been found to have a main role in the treatment of various illnesses such as upper respiratory tract infections, and alcoholic liver diseases can be extracted from natural extracts like citrus fruits, apple, onion, parsley, berries, green tea, and red wine, rutin extracted from the legume (*Sophora japonica Linn*.). It has been found to effectively work in the treatment of different types of thyroid dysfunction. An *in vivo* study was conducted on mice to ensure its effectiveness. *In vivo*, studies include investigations in hepG2 cells, fisher rat thyroid cell line FRTL-5 thyroid cells, and thyroid type 1 iodothyronine deiodinase activity [186,228]. Apart from these, it has been majorly investigated to know about its impact via clinical trials which includes the study of the pharmacokinetics of quercetin supplementation and treatment of upper respiratory tract infections [229,230].

### Baicalein

The baicalein can be extracted from the roots of *Scutellaria baicalensis* and *Scutellarialateriflora*, olive leaves, etc., and is known to have an efficient effect on profound illnesses which include thyroid cancer, Type 2 diabetes, breast cancer and numerous other types of cancers [231,232]. Studies on rat intestinal α-glucosidase, female mice, and breast cancer cell transplantation tumor models have been conducted to ensure its therapeutic efficacy. Also, its efficacy has been investigated *in vitro* in PR-B and T47D cells, OVCAR5 cells, MCF-7 and MDA-MB-231 breast cancer cells, thyroid type 1 iodothyronine deiodinase activity [233,234].

### Sesamin

This phytochemical could be extracted from sesame seeds. It has been known to showcase its effect on thyroid cancer and Type 2 diabetes. A clinical trial was conducted on 48 patients to ensure the sesamin effect on Type 2 diabetes. *In vivo*, studies on adult female albino diabetic rats have been done along with *in vitro* studies on thyroid cancer cell lines (FTC-133). These studies showed its potential to treat thyroid malfunctions along with its major impacts on other illnesses such as diabetes [235].

### Warfarin

The major sources of warfarin include sweet clover and tonka beans. In a clinical trial on patients upon concomitant administration of amiodarone and warfarin in hyperthyroid patients, patientswith Graves’ disease, and patients with nonrheumatic atrial fibrillation, have shown its proven therapeutic efficacy for the treatment of warfarin-induced hypoprothrombinemia to know its vast impacts on people suffering from hypoprothrombia, non-rheumatic atrial fibrillation [236,237].

### Delphinidin

Pigmented vegetables and fruits, particularly blueberry are the main sources of this phytochemical namely, delphinidin. Various *in vitro* studies like investigations on Human CRC cell lines, human thyroid cancer cells, HCC cells, NSCLC cells, HepG2 cells, human liver carcinoma cell lines, osteosarcoma cell lines, etc. have been subjected to determining its therapeutic efficacy for the treatment of diseases like thyroid cancer, liver carcinoma, osteosarcoma, etc [238,239].

### Butin

It can be extracted from *Acacia mearnsii, Vernonia anthelmintica*, and *Dalbergia odorifera*. It has showcased major roles in the treatment of thyroid cancer. An in vitro study in SW579 cells in it reduces proliferation, promotes apoptosis, limits motility, and causes mitochondrial damage in SW579 cells by up-regulating Bax and down-regulating Bcl-2 and Bcl-xL protein expression. Butin was used to reverse the effects of RhoBTB2 on SW579 cells [240].

### Piperine

*Piper nigrum* fruits, *Piper longum Linn* are the major sources of piperine. *In vivo*, studies on adult male swiss albino mice, mice, male Wistar rats were undertaken to ensure its importance in the treatment of thyroid-relevant manifestations. In a study, Panda et al investigated that piperine reduces thyroid hormone levels, glucose levels, and hepatic 5′ D activity in mature male mice [240]. In another study, Gupta et al studied the effect of piperine which shows that it inhibits the FFA-induced TLR4-mediated inflammation and enrichment of acetic acid-induced ulcerative colitis in mice [241]. Piperine not only was investigated to showcase therapeutic actions against thyroid-relevant dysfunctions but rather it has many efficacious roles in various other illnesses also [242]. In an investigation, the different aspects of piperine inculcated by Vijaykumar et al*.* into which they investigated in hyperlipidemic rats, piperine is an active component derived from *Piper nigrum*, to alter hormonal and apolipoprotein profiles [243].

### Apigenin

Apigenin is a flavonoid that is prevalent in vegetables and fruits and possesses anti-inflammatory, antioxidant, and anticancer effects. It has been found to have therapeutic actions against the thyroid relevant complications as investigated by Panda et al. in their investigation in which apigenin has the ability to control diabetes, thyroid dysfunction, and lipid peroxidation caused by various pathogens [244]. In another study, Zang et al. studied apigenin’s anti-neoplastic actions on the BCPAP cell line of papillary thyroid cancer (PTC) in which they investigated that apigenin reduces BCPAP cell viability in a dose-dependent manner [245].

### Myricetin

Myricetin is a polyphenolic molecule that belongs to the flavonoid family and has antioxidant effects. V egetables (including tomatoes), nuts, berries, fruits (including oranges), and tea are all common dietary sources of it. Various studies have been conducted to inculcate the importance of myricetin in diseases like thyroid malfunctions. An *in vivo* investigation conducted by Jo et al., examined SNU-80 HATC cells and treated these with myricetin (various concentrations), and compared it with untreated controls. They found that myricetin is a strong inducer of HATC cell death. Hence, it might be beneficial in the development of HATC therapeutics [246].

### Curcumin

Curcumin is a brilliant yellow substance that is generated by *Curcuma longa* plants. The main curcuminoid in turmeric (*Curcuma longa*), belongs to the Zingiberaceae ginger family. Enormous *in vitro* and *in vivo* studies have been investigated to acknowledge its efficacy and it has been proven to be the most prominent therapeutic agent for not only thyroid-relevant disorders but also for various other diseases. A study was conducted by Esposito et al. in which they investigated the inhibitory effect of curcumin on TPC1 thyroid cell lines [247]. Another study was conducted by Perna et al which they studied the effects of curcumin on the TPC1 papillary thyroid cancer cell line in which they concluded that curcumin extract treatment has anti-inflammatory and antioxidant characteristics, as well as the ability to alter cell cycle with somewhat varied effects depending on the extract. Curcumin can also affect cell metabolic activities [248].

## Post-COVID-19 complications

**Figure 8:**
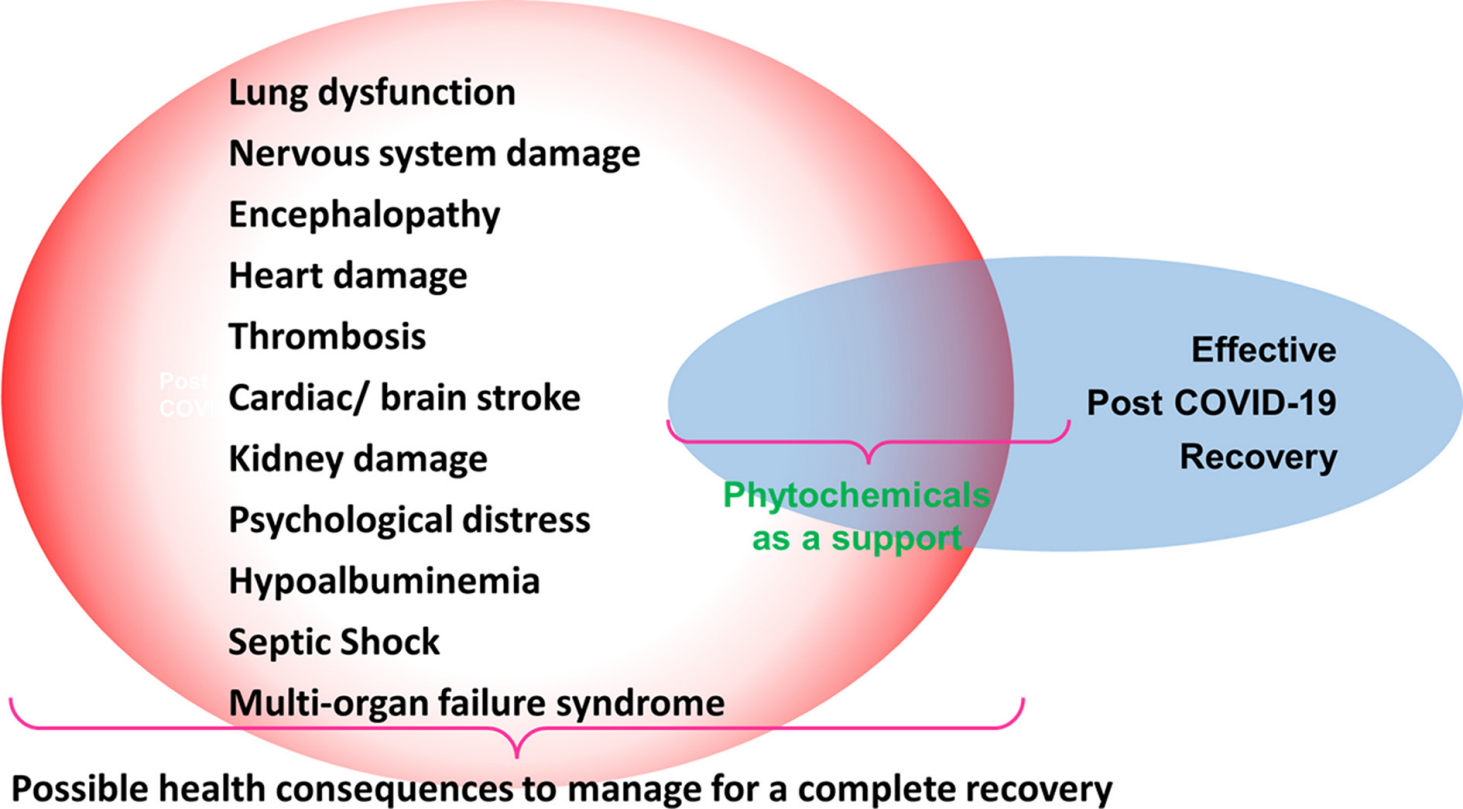
COVID-19 affects various organs of the body and post-COVID-19 complications cause’ further damage Possible consequences of COVID-19 disorders and phytochemical mediated effective recovery

Nervous system damage, lung, liver, kidney, heart damage, thrombosis, cardiac/brain stroke, encephalopathy, and psychological distress are all late COVID-19 consequences. Other problems, such as hypoalbuminemia, septic shock, and multi-organ failure syndrome have also been described in several studies. Figure 8 depicted certain disorders that resulted in post-COVID-19 complications. Further, the ARDS and cytokine storm after severe COVID-19 infection is the major cause of COVID-19-related death. Pro-inflammatory cytokines for instance interleukin IL-1, IL-2, IL-6, IL-8, IL-17, IFN-γ, and TNF-α were raised in these patients, affecting clinical symptoms and severity [249]. The phytochemical-based treatment can be a safer and more cost-effective option to deal with the post-COVID-19 complications mediated by a cytokine storm.

Table 2 enlisted phytochemicals which can be successfully exploited for the treatment of post-COVID-19 complications in a person already suffering from other disorders. For instance, recently nicotine which is a cholinergic agonist works by activating the 7-nAChR receptor. It also inhibits pro-inflammatory cytokines like IL-1, IL-6, and TNF-α, but not anti-inflammatory cytokines like IL-10 [250]. Nicotine, when taken in the form of gum, inhalers/sprays, or patches, may be helpful in the treatment of severe withdrawal symptoms [251,252]. In this section, we provided brief information about certain complications that occurred as a result of COVID-19 infection and are responsible for high mortality among the infected populations.

COVID-19 survivors have been reported with gastrointestinal and hepatobiliary complications. COVID-19 exhibits severe viral fecal shedding. After the commencement of SARS-CoV-2 infection symptoms, detectable viral RNA lasts an average of 28 days, and it lasts an average of 11 days after negative respiratory specimens. COVID-19 has the potential to alter the gut microbiota by enriching opportunistic infectious organisms and encouraging the consumption of beneficial eaters. Influenza and other respiratory infections have previously discovered the ability of gut flora to modify the course of respiratory diseases (intestinal lung axis) [253]. In COVID-19, the anaerobe *Faecali bacterium prausnitzii*, which generates butyrate and is normally associated with good health is adversely connected with illness severity. COVID-19’s long-term effects on the gastrointestinal tract, particularly post-infection irritable bowel syndrome, and dyspepsia, have now been studied. Obesity is a critical risk factor for major cases of infectious illnesses such as influenza, hepatitis, and hospital infections [254,255].

The illnesses such as tuberculosis, community-acquired pneumonia, and sepsis have better clinical effects: Obese adults are more likely to be obese than non-obese individuals. In support of the ‘obesity paradox’, the physiological response to infection will be influenced by the core elements of the obesity hypothesis. Obesity, like influenza infections, appears to make COVID-19 more severe. Obesity is a metabolic condition marked by alterations in systemic metabolism such as insulin resistance, elevated blood glucose, adiponectin alterations e.g., increased leptin and decreased adiponectin, and persistent low-grade inflammation [256,257]. The causes of hormonal and nutritional problems are well-documented. Obese people have a weakened immune system, making them more susceptible to infection. Hyperglycemia is a significant symptom of Type 2 diabetes and is strongly linked to obesity. Uncontrolled serum glucose has been proven to enhance the death rate of COVID-19 substantially. [258] Uncontrolled blood glucose can affect immune cell function directly or indirectly during infection by producing oxidants and glycosylation products [259]. Similarly, insulin and leptin signaling increase the generation of effector cytokines like IFN-γ and TNF-α by positively regulating cellular glycolysis and boosting the inflammatory response of T cells [260,261]. These metabolic parameters interact and influence immune cell metabolism, and influence the pathogen like SARS-CoV-2's functional response [262].

The inflammatory response can also be influenced by dietary fatty acids. Long-chain fatty acid-derived prostaglandins are acute pyrogens that can cause local inflammation during infection. Through cyclooxygenase (COX) activity, omega-3 polyunsaturated fatty acids can promote anti-inflammatory responses, while omega-6 fatty acids can moderate the pro-inflammatory generation of COX prostaglandins [263,264]. In obese people, fatty acid derivatives can have a direct effect on COVID-19. Preclinical evidence suggests that fatty acid-derived high-resolution lipid mediators have some effect, since they may not be sufficient for obese people, and hence cannot adequately resolve the inflammatory response during infection [265]. Other fatty acids, such as cholesterol, are required for life. SARS-CoV-2 attaches to the protein S on the cell’s ACE2 receptor and uses cholesterol to stimulate virus germination and spread to other cells. The lowering of cholesterol in cells expressing ACE2 results in a considerable decrease in viral protein S binding [266]. Obesity also enhances the severity of COVID-19 in patients with metabolic fatty liver disease, and the obesity rate of obese adults is more than 6 times that of obese adults.

There is a risk of severe COVID-19 regardless of age, gender, or comorbidities such as high blood pressure, diabetes, or dyslipidemia [267]. Obese patients’ physical attributes may further raise the severity and risk of COVID-19. Obstructive sleep apnea and other respiratory disorders increase the risk of pneumonia in obese people, which is linked to hypoventilation, pulmonary hypertension, and high blood pressure [268]. The focus of hospitals is to undertake supportive treatments such as intubation, which is heightened by a big waist circumference and weight gain [269]. Therefore, the prognosis of obese individuals with COVID-19 may be exacerbated by the higher clinical care load of this group of patients who are already vulnerable. The severity of COVID-19 problems has been demonstrated to be affected by age and gender. Men are more prone than women to develop significant sequelae from SARS-CoV-2 infection [269].

In order to determine the prevalence of persistent symptoms, complications, and any risk factors related to COVID-19, a single center, prospective, observational study was conducted in a tertiary respiratory care institute in North India from June 2021 to August 2021 with 224 cases of previously treated COVID-19/ongoing symptomatic COVID-19 (those patients who were manifesting symptoms beyond 4 weeks). Risk factors and outcome variables were analysed using both univariate and multivariate methods. AUC was calculated after performing ROC on the predictor variables. A *P*-value of 0.05 or less was regarded as significant. The most prevalent symptom among the 24.6% of patients with symptoms at follow-up was fatigue (51.8%), followed by dyspnea (43.8%) and anxiety (43.3%). The analysis revealed that fibrosis (15.2%) was found to be the most prevalent COVID-19 consequence, followed by pulmonary thromboembolism (PTE) (12.1%), echocardiographic abnormalities (11.2%), and pulmonary mucormycosis (5.4%). As independent risk factors for problems following COVID-19, female gender, the presence of comorbidities, and the need for non-invasive or invasive breathing during hospitalization appeared [270]. This study highlights the significant morbidity cost that COVID-19 imposed on patients who appeared to be healed and enumerates the risk factors linked to sequelae and symptom persistence. The improved organization of medical resources and harmonize follow-up care given to COVID-19 patients resulted in better disease management.

In another study, 100 post-COVID patients with extreme fatigue (aged 18 to 70) symptoms were enrolled. 50 of them received care at home, while 50 received care at a hospital. The automated analyzer was used to measure the serum TSH and FT4 levels. TSH and FT4 levels in patients receiving care at home and those in a hospital were 1.59 and 2.96 mIU/L and 9.27 and 17.28 pmol/L, respectively. There was a significant difference in the level of blood TSH (*P=*0.0001) and FT4 (*P=*0.0001) in both groups and it was inferred that patients treated at home for post-COVID fatigue had shown increased serum TSH and FT4 levels than individuals treated in hospitals [271]. The observation suggests a better healthy homely environment helps to overcome thyroid-related complications.

## Challenges and future outlook

Medicinal herbs and natural products-based formulations have a lot of potentials and can be used against fighting diseases [272,273]. Plants have their own outstanding and highly effective defense mechanisms and antipathogenic defense systems which can be exploited in the field of drug design [274]. Despite of plethora of studies in this field, the actual potential of plant-originated phytochemicals against various disease conditions remains unexplored. In this context, when dealing with complicated diseases, more and more research is required for locating active medicinal plants, identifying active chemical constituents, and investigating potential synergistic effects at the molecular level. Further, the chemistry of the active substances in plant extract must need to be investigated. Appropriate preclinical, toxicological, and clinical investigations should be warranted while exploring the therapeutic potential of phytochemical-based formulations. One of the hurdles while using plant phytochemicals is the low yield of desired molecules while extraction which can be resolved by establishing sustainable technology for the efficient production of desired molecules economically at cheap prices. However, there is still a long way to go in terms of developing more evidence-based phytotherapy or incorporating highly active natural compounds into clinical practice.

In this regard, the groups of biologists, pharmacognosy personnel, natural product chemists, synthetic chemists, toxicologists, physicians, and government officials, working in close association would pave the way for the development of plant-based therapeutics in a more efficient and scientifically proven way. The mining of antipathogenic ingredients from a vast pool of natural herbs needs a holistic approach [272]. Moreover, the growing demand for exotic medications and supplements for sustainable healthcare required bulk production setup with proper scientific assessment of the quality, safety, pharmacological effects, and indexing therapeutic benefits of phytoconstituents. The strategies for medicinal plant production and outsourcing as quickly as possible is needs to be worked out. As SARS-CoV-2 continues to evolve, new variants with drug resistance keep emerging. The advantages of phytochemicals are polypharmacology and broad-spectrum antiviral activity. Therefore, they can be combined with direct-acting antivirals to achieve synergistic antiviral effects and suppress drug resistance. For instance, recent studies reported several drug-resistant hot spots that discuss probable clinical signs of Paxlovid resistance. In this context, multiple therapeutic targets of infectious agents can be targeted by phytochemicals which would have the potential to resolve the drug resistance problem efficiently [275,276].

Many popular herbal substances appear to be in higher demand, such as the demand for plants like *Echinacea* is skyrocketing, but current supply methods can’t manage to keep up. This raises serious concerns regarding the integrity of the supply chains involved in the manufacturing of these plant-based healthcare items [272–274]. The obstacles are numerous while exploring phytochemicals, ranging from growing consumer demand to those relating to the health of workers along with value product supply chains. The impact of the current COVID-19 pandemic further influences the process of exploring the potential benefits of plant-based medicine due to various conditions imposed therein. As a result of this worldwide problem, not only the breeding and manufacture of material get affected, but also the delivery of authentic high-quality final products gets hampered and requires considerably more attention [272,273].

**Figure 9:**
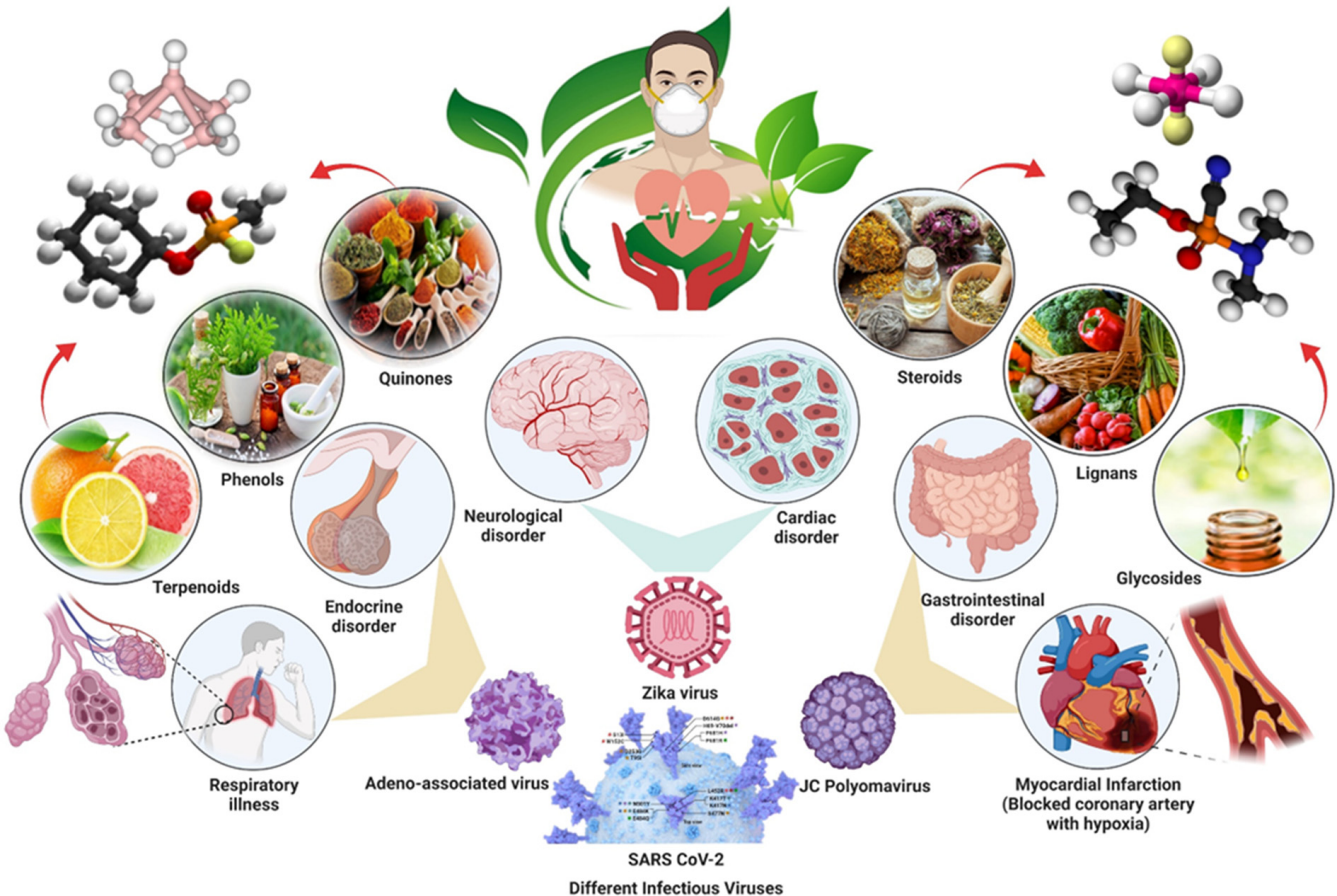
Phytochemical-based formulations as a therapeutic option for futuristic viral infections and associated anomaly to the multiple organ systems A schematic illustrates futuristic usage of potential phytochemicals against various types of viral infections.

The holistic approach for better science, better patient care management, and a healthier planet is the need of an hour [2]. The illustration of phytochemical-based therapeutic formulations as an option for present and futuristic infectious diseases has been shown in Figure 9.

The government and private funding agencies must make funds available to ensure that the potential of natural products and herbal medical items would be thoroughly investigated. The main limitations of this comprehensive evaluation stem from the research that has been conducted on these still-experimental medications are varied. Many of such studies examined, had a limited number of patient enrollments, resulting in data that was not statistically significant. Recently, numerous classic medications with well-known mechanisms of action are being re-proposed for COVID-19 treatment. However, the emergence of SARS-CoV-2 variants such as Omicron and Delmicron and the effects of phytochemicals against these mutants needs special attention and should be explored [3–5]. Furthermore, the assessments of phytomedicines must consider the circumstances of specific patients, such as children and pregnant women, who are excluded from clinical trials due to ethical concerns. The present review's strength is that it provides current data on the management of COVID-19 in a setting where knowledge is continuously changing, and growing enormously, hence needs to be available to all in a precise manner which will be useful to health professionals for proper decision making.

## Concluding remark

COVID-19 caused by the SARS-CoV-2, has created a worldwide disaster that has touched many facets of human existence. The virus mostly affects the lungs, but it can also affect the kidneys, the stomach, and various endocrine glands including the thyroid. Viral infections are important environmental factors in the development of autoimmune thyroid diseases and subacute thyroiditis, among other thyroid disorders. Considering such illnesses in this review we have focused on the role of phytochemicals either alone or in combination with patients suffering from thyroid-relevant disorders with COVID-19 complications. Viruses, along with their accompanying inflammatory-immune responses, maybe a key predictor of lifelong thyroid function, aiding in the determination of an individual’s thyroid biography’. Researchers seek innovative antiviral formulations due to the insufficiency of targeted medications and vaccines, as well as the pandemic of COVID-19 illness caused by SARS-CoV-2 associated with thyroidal dysfunctions. The viral infection of the thyroid gland, as well as the immuno-inflammatory responses that accompany it, are known to interact in a complex fashion. As a result, the SARS-CoV-2 might have hitherto unknown significant effects on thyroid function. Phytochemicals have shown an efficacious role in the treatment of thyroid and various other thyroid-related disorders.

However, the therapeutic efficacy of these phytochemicals-based formulations needs to be validated in terms of the mechanism by which they can prevent organ dysfunction. A deeper insight and solutions on how the aforementioned phytochemicals should be utilized towards better management of not only the current COVID-19 pandemic but any futuristic pandemic needs to be further elucidated. In this context, the role of quercetin, apigenin, myricetin, tryptophane, L-Arabinose, and curcumin have shown immunomodulatory and viral inhibitory potential. Therefore, these phytochemicals can be used as potential therapeutic drugs for the treatment of COVID-19-related thyroid dysfunction and post-COVID-19 complications. Furthermore, these phytochemicals-based formulations showed various therapeutic properties not only against modulating thyroid gland function, but they tend to bind main protease site of the SARS-CoV-2 and inhibit viral infection, and help in curbing the infection. Considering the advantages offered by phytochemicals as a safer and cost-effective medication that hss the potential to deal with the current COVID-19 pandemic-associated comorbidities. Taken together, the content of this review will provide readers not only the mechanistic insight into COVID-19 and its effect on thyroid function but also simultaneously provide information about the phytochemical-based therapeutics for the treatment of thyroid dysfunction and post-COVID-19 complicatios.

## Declarations

We confirm that this manuscript is an original work and is not under consideration by any journal.

## Abbreviations

3CLpro: 3-chymotrypsin like protease
ACE: angiotensin-converting enzyme
ACE2: angiotensin-converting enzyme -2
AChE: acetyl choline esterase
AChE1: acetyl choline esterase 1
ARB: angiotensin receptor blockers
ARDS: acute respiratory distress syndrome
AST: aspartate amino transferase
BDMC: bidemethyloxycurcumin
CNS: central nervous system
CoV: coronavirus
COVID-19: coronavirus disease 2019
COX-2: cyclooxygenase-2
CRP: C-reactive protein
CTRI: Clinical Trial Registry-India
DIC: diffuse intravascular coagulation
DM2: diabetes miletus type-2
DMC: dimethoxy curcumin
DNA: deoxyribonucleic acid
ECG: electrocardiogram
EGCG: epigallacto-catechin-3-gallate
ENT: ear nose throat
ESR: erythrocyte sedimentation rate
ESS: euthyroid sick syndrome
FT3: free triiodo thyroxine
FT4: free thyroxine
FTH: ferretin heavy
FTP: failure to progress
GCG: gallo catechin gallate
GGO: ground glass opacity
Hb: hemoglobin
HCoV-OC43: human coronavirus OC43
HCoVs: human coronavirus
HCRT: high-resolution computed tomography
HPG: hypothalamus pituitary gonad
IC: inspiration capacity
ICU: Intensive Care Unit
IFN: interferon
IL: interleukin
Inos: inducible nitric oxide synthase
LH: leutinizing hormone
M: molar
MAPK: mitogen-activated protein kinase
MERS-CoV: Middle East Respiratory Syndrome Coronavirus
MHC: major histocompatibility complex
MMPs: matrix metalloproteinases
Mpro: main protease
MRI: magnetic resonance imaging
MTT assay: (3-(4,5-dimethylthiozolyl-2)-2,5-diphenyltetrazolium bromide assay
NF-κB: nuclear factor-κB
NTI: non-thyroidal illness syndrome
PC: parafollicular cells
PCR: polymerase chain reaction
PLpro: papain-like protease
PTC: papillary thyroid cancer
RAAS: renin–angiotensin aldosterone system
RAS: renin–angiotensin system
RBD: receptor-binding domain
RD cell: rhabdomyosarcoma cell
ROC curve: receiver operating characteristics
S protein: spike glycoprotein
SARS: severe acute respiratory syndrome
SARS-CoV: severe acute respiratory syndrome coronavirus
SAT: subacute thyroiditis
SCoV: SARS-associated coronavirus
SpO2: oxygen saturation
sVNT: surrogate virus neutralization test
T: testosterone
TMPRSS2: transmembrane serene protease 2
TNF-α: tumor necrosis factor-α
TPO: anti-thyroid peroxidase
TRAb: anti-TSH receptor antibodies
TSH: thyroid stimulating hormone
TT3: Total T3
TT4: Total T4
US: ultrasound
VOC: variants of concern
VOI: variants of interests
VUM: variants under monitoring
WHO: World Health Organization
